# Phase-transition nanodroplets with immunomodulatory capabilities for potentiating mild magnetic hyperthermia to inhibit tumour proliferation and metastasis

**DOI:** 10.1186/s12951-023-01885-4

**Published:** 2023-04-17

**Authors:** Qiaoxi Qin, Yang Zhou, Pan Li, Ying Liu, Ruxi Deng, Rui Tang, Nianhong Wu, Li Wan, Ming Ye, Hong Zhou, Zhiming Wang

**Affiliations:** 1 Department of Ultrasound, Affiliated Hospital of Southwest Jiaotong University, The Third People’s Hospital of Chengdu, Chengdu, 610031 China; 2 Institute of Biomedical Engineering, College of Medicine, Southwest Jiaotong University, Chengdu, 610031 China; 3 Department of Ultrasound, The Second Affiliated Hospital of Chongqing Medical University, Chongqing, 400010 China; 4 Institute of Ultrasound Imaging of Chongqing Medical University, Chongqing, 400010 China; 5 Department of Health Management (Physical Examination) Center, The Second Affiliated Hospital of Chongqing Medical University, Chongqing, 400010 China

**Keywords:** Mild magnetic hyperthermia, Immunogenic cell death, Phase transition, Cavitation effect, Immune adjuvant

## Abstract

**Background:**

Magnetic hyperthermia (MHT)-mediated thermal ablation therapy has promising clinical applications in destroying primary tumours. However, traditional MHT still presents the challenges of damage to normal tissues adjacent to the treatment site and the destruction of tumour-associated antigens due to its high onset temperature (> 50 °C). In addition, local thermal ablation of tumours often exhibits limited therapeutic inhibition of tumour metastasis.

**Results:**

To address the above defects, a hybrid nanosystem (SPIOs + RPPs) was constructed in which phase transition nanodroplets with immunomodulatory capabilities were used to potentiate supermagnetic iron oxide nanoparticle (SPIO)-mediated mild MHT (< 44 °C) and further inhibit tumour proliferation and metastasis. Magnetic-thermal sensitive phase-transition nanodroplets (RPPs) were fabricated from the immune adjuvant resiquimod (R848) and the phase transition agent perfluoropentane (PFP) encapsulated in a PLGA shell. Because of the cavitation effect of microbubbles produced by RPPs, the temperature threshold of MHT could be lowered from 50℃ to approximately 44℃ with a comparable effect, enhancing the release and exposure of damage-associated molecular patterns (DAMPs). The exposure of calreticulin (CRT) on the cell membrane increased by 72.39%, and the released high-mobility group B1 (HMGB1) increased by 45.84% *in vivo*. Moreover, the maturation rate of dendritic cells (DCs) increased from 4.17 to 61.33%, and the infiltration of cytotoxic T lymphocytes (CTLs) increased from 10.44 to 35.68%. Under the dual action of mild MHT and immune stimulation, contralateral and lung metastasis could be significantly inhibited after treatment with the hybrid nanosystem.

**Conclusion:**

Our work provides a novel strategy for enhanced mild magnetic hyperthermia immunotherapy and ultrasound imaging with great clinical translation potential.

**Supplementary Information:**

The online version contains supplementary material available at https://doi.org/10.1186/s12951-023-01885-4.

## Introduction

Although remarkable advances have been achieved in conventional oncologic methodologies, including surgery, radiotherapy and chemotherapy [1], malignant tumours remain a threat to public health as one of the dominant causes of mortality worldwide [2]. The need for innovative and effective cancer treatments with substantially improved therapeutic effects and reduced side effects remains. On account of its unique advantages over conventional oncologic methodologies (minimal invasiveness, inferior mutagenic potential, fewer side effects and improved tumour specificity, etc. [3, 4]), hyperthermia therapy (HTT) has emerged as a potential strategy for tumour treatment. HTT is a treatment mode in which the temperature of targeted tumour sites is raised above 41 °C by virtue of photothermal agents or magnetic-thermal conversion agents, corresponding to photothermal therapy (PTT) and magnetic hyperthermia (MHT), respectively. Nanomaterial-based MHT can treat local tumours utilizing the heat generated by magnetic nanoparticles under an alternating magnetic field (AMF). Unlike PTT, which is limited by laser penetration capacity, MHT exhibits superior penetration with no tissue-depth attenuation of the magnetic responsiveness of magnetic nanoparticles under AMF, rendering MHT a competitive therapeutic candidate for deep and inaccessible tumours [5–7]. However, traditional MHT at high temperatures (> 50 °C) can eradicate primary tumours effectively but may ruin tissue-resident immune cells and tumour-associated antigens, which are vital to the immune response against cancer [8, 9]. High temperatures may also cause great discomfort for patients and lead to serious damage to neighbouring normal organs or tissues owing to inevitable heat diffusion [10]. In recent years, the concept of mild MHT (~ 43 °C) has been recognized for its clinical promise for cancer treatment [11–15]. Around this temperature, heat cytotoxicity is markedly reduced, while safety and comfort are improved [16]. Nevertheless, currently, there are still a few limitations of mild MHT alone, including time-consuming treatment duration, the need for repeat treatments, limited tumour cell inhibition, or inadequate antigen release or exposure [14, 15].

To address the abovementioned problems, it is worth seeking an effective way to enhance the efficiency of mild MHT. The synergistic anticancer effect of microbubbles has drawn researchers’ attention, focusing on the applications of synergistic therapy with ultrasound (low-intensity focused ultrasound [17, 18], high-intensity focused ultrasound [19, 20]) or laser (ultrafast NIR-laser-based microsurgery [21], PTT [22, 23]), while the application of microbubbles in combination with MHT has been rare. The instant expansion and explosion of microbubbles may produce a microcavitation effect that causes localized mechanical damage to surrounding cells or tissues, which has been proven to facilitate the destruction of cancer cells in synergistic therapies [24–27]. However, traditional microbubbles suffer from the drawbacks of large particle size (in the range of several to hundreds of micrometres), instability, and short blood circulation time [28]. Compared with traditional microbubbles, PLGA liquid‒gas phase-transition nanodroplets may be an advantageous candidate in cancer treatments for their nanoscale size and stability, which allows them to “squeeze” through pores in the endothelial gap or to permeate the cell membrane more easily. The outstanding magnetic-thermal conversion property of the hybrid nanosystem provides appropriates condition for the phase transition of liquid‒gas phase-transition nanodroplets, which means that once exposed to the thermal stimulation mediated by mild MHT, the nanodroplets can be converted into gas microbubbles, exhibiting a special form of magnetic droplet vaporization (MDV) [29]. Thus, PLGA liquid‒gas phase-transition nanodroplets are expected to act as an optional strategy for potentiating mild MHT that can not only compensate for the underlying lack of efficiency of mild MHT alone but also avoiding the risk of high temperature. Moreover, the liquid‒gas phase-transition nanodroplets possessed the function of drug loading, and the collapse of the microbubbles helps to accelerate the release of the drug contained in the carrier during the phase transition [30, 31]. The microbubbles produced by phase-transition nanodroplets through MDV can endow the carrier with certain ultrasonic imaging properties [32, 33]. These properties lay the foundation for the integration of diagnosis and treatment of phase-transition nanodroplets. Although the combination of mild MHT and liquid‒gas phase-transition nanodroplets holds promise for suppressing the growth of primary tumours, it is not effective in preventing tumour metastasis or recurrence.

In recent decades, cancer immunotherapy has emerged as the most promising approach to defeat cancer, which is reflected not only in the suppression of primary tumour growth but also, more importantly, in the prevention of tumour metastasis or recurrence by harnessing the inherent immune system of patients [34, 35]. However, the low immunogenicity of a majority of tumours is often the first obstacle to the activation of the immune system [36]. Based on mild MHT, the liquid‒gas phase-transition nanodroplets facilitate the immunogenic cell death (ICD) of tumour cells, and the cavitation effect-mediated cell membrane disintegration leads to increased release and exposure of DAMPs [37], which includes passively released high mobility group B1 (HMGB1), actively secreted adenosine triphosphate (ATP), and surface-exposed calreticulin (CRT) [38], thus turning “cold” tumours into “hot” ones, reversing the immunosuppressive tumour microenvironment (TME) and sensitizing tumours to immunotherapy [39–42]. Resiquimod (R848), a potent Toll-like receptor 7 and 8 (TLR-7/8), is considered an immune adjuvant that can improve the body’s immune responses to antigens [43, 44]. Accompanied by R848, MHT-associated DAMPs can be recognized by Toll-like receptors (TLRs), and the stimulation of TLRs leads to the activation of dendritic cells (DCs), upregulating their costimulatory factors CD80 and CD86, which are the hallmarks of mature DCs, converting their phenotype from immunosuppressive to immunogenic [45, 46]. Matured DCs acquire enhanced antigen cross-presentation ability [47–49] and then secrete multiple proinflammatory cytokines, including interleukin 6 (IL-6), including interleukin 12 (IL-12), and tumour necrosis factor α (TNF-α), regulating antitumour immune responses [50], which facilitates the activation of CTLs (cytotoxic T lymphocytes) to eradicate tumours [51].

**Fig. 1:**
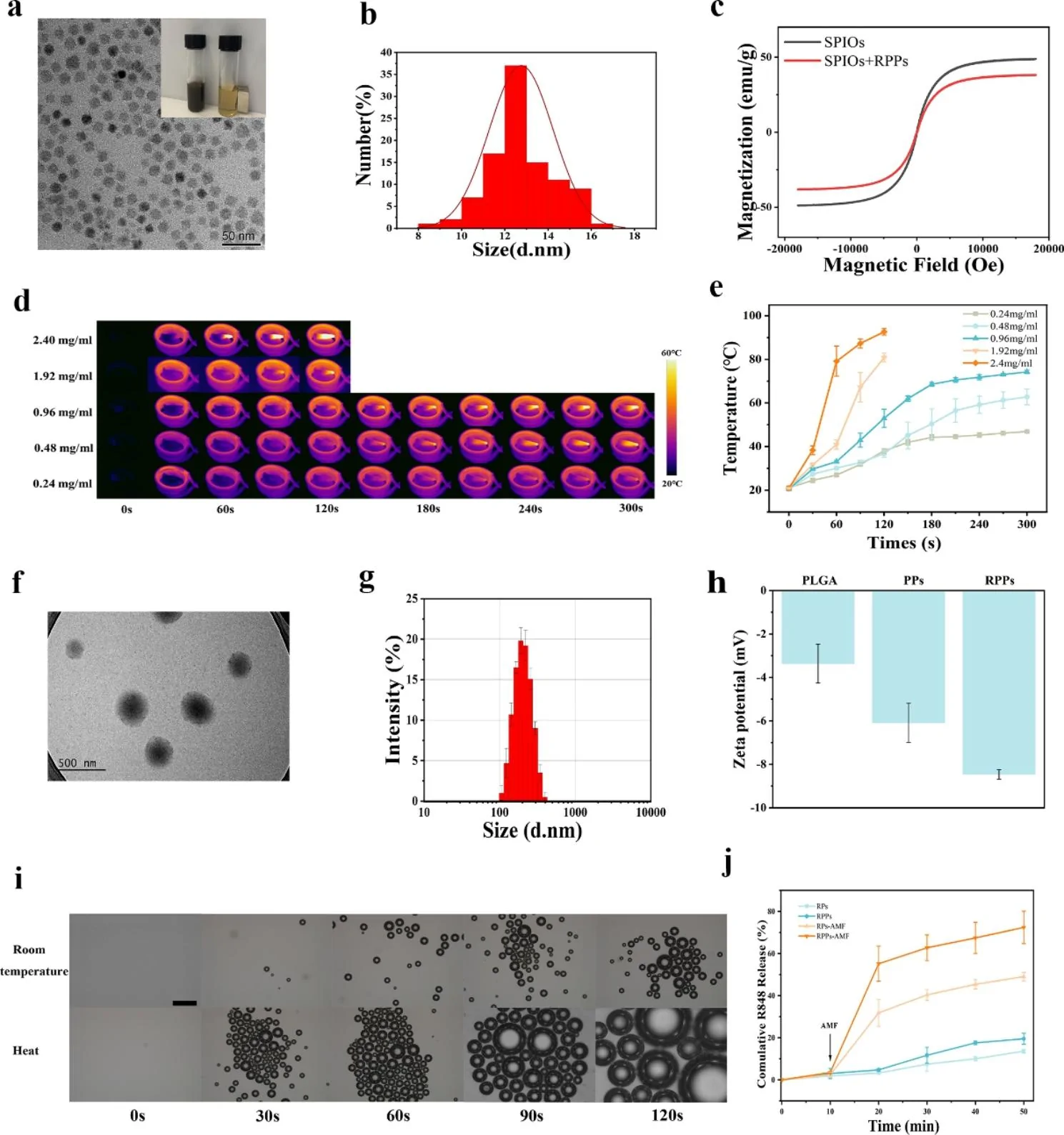
Characterizations of SPIOs and RPPs. **a**) TEM of SPIO inset: SPIO dispersion (left) and under an external magnetic field (right). **b**) Size distribution of SPIOs. **c**) Magnetic hysteresis loop of SPIOs and SPIOs + RPPs. **d**) Real-time *in vitro* IR thermal imaging of SPIOs at different concentrations. **e**) Quantitative temperature rise curves of SPIOs at different concentrations. **f**) TEM of RPPs. **g**) Hydrodynamic size of RPPs. **h**) Zeta potential of PLGA, PPs, and RPPs (n = 3). **i**) Droplet vaporization of RPPs *in vitro* observed by optical microscopy. Scale bar = 50 μm. **j**) The release profiles of RPs and RPPs with or without AMF in 10 min

In the context of these interesting findings, in this work, we designed a hybrid nanosystem (SPIOs + RPPs) to potentiate mild MHT to inhibit tumour proliferation and metastasis. As illustrated in Fig. 1a, supermagnetic iron oxide nanoparticles (SPIOs) were prepared by the coprecipitation method and showed excellent magnetothermal conversion performance. R848 served as the immune adjuvant to further activate antigen presenting cells (APCs) [52]. PFP with a low boiling point of 29 ℃ was incorporated as a phase transition agent [53] by encapsulation inside the PLGA shell through a typical double-emulsion process (w/o/w), which ultimately formed magnetic-thermal sensitive phase-transition nanodroplets, named R848-PFP@PLGA(RPPs). As shown in Fig. 1b, when the primary tumour was injected with SPIOs + RPPs, the temperature increased locally and remained below 44 ℃ to implement mild MHT. Under magnetic thermal stimulation, the nanodroplets underwent a liquid-to-gas transition and generated microbubbles in situ. With the combination of MHT andn cavitation effect, an enhanced ICD was generated, accompanied by more HMGB1 and ATP release and more CRT exposure. Moreover, magnetic-thermal stimulation of the phase transition could be conducive to R848 release. Ultimately, bioactive DAMPs in combination with R848 adjuvant created a highly immunogenic TME, thus effectively reversing the immunosuppressive TME and activating DCs and CTLs to combat tumour proliferation and metastasis. Moreover, microbubbles can also act as contrast agents, endowing the nanosystem with the function of ultrasound imaging, which provides the potential for guidance or monitoring during treatment.

Thus, our work not only presents the hybrid nanosystem (SPIOs + RPPs) as a promising therapeutic candidate with great vaccine-like function for potentiating mild magnetic hyperthermia immunotherapy but also holding great potential for cancers theranostics applications.

**Scheme 1:**
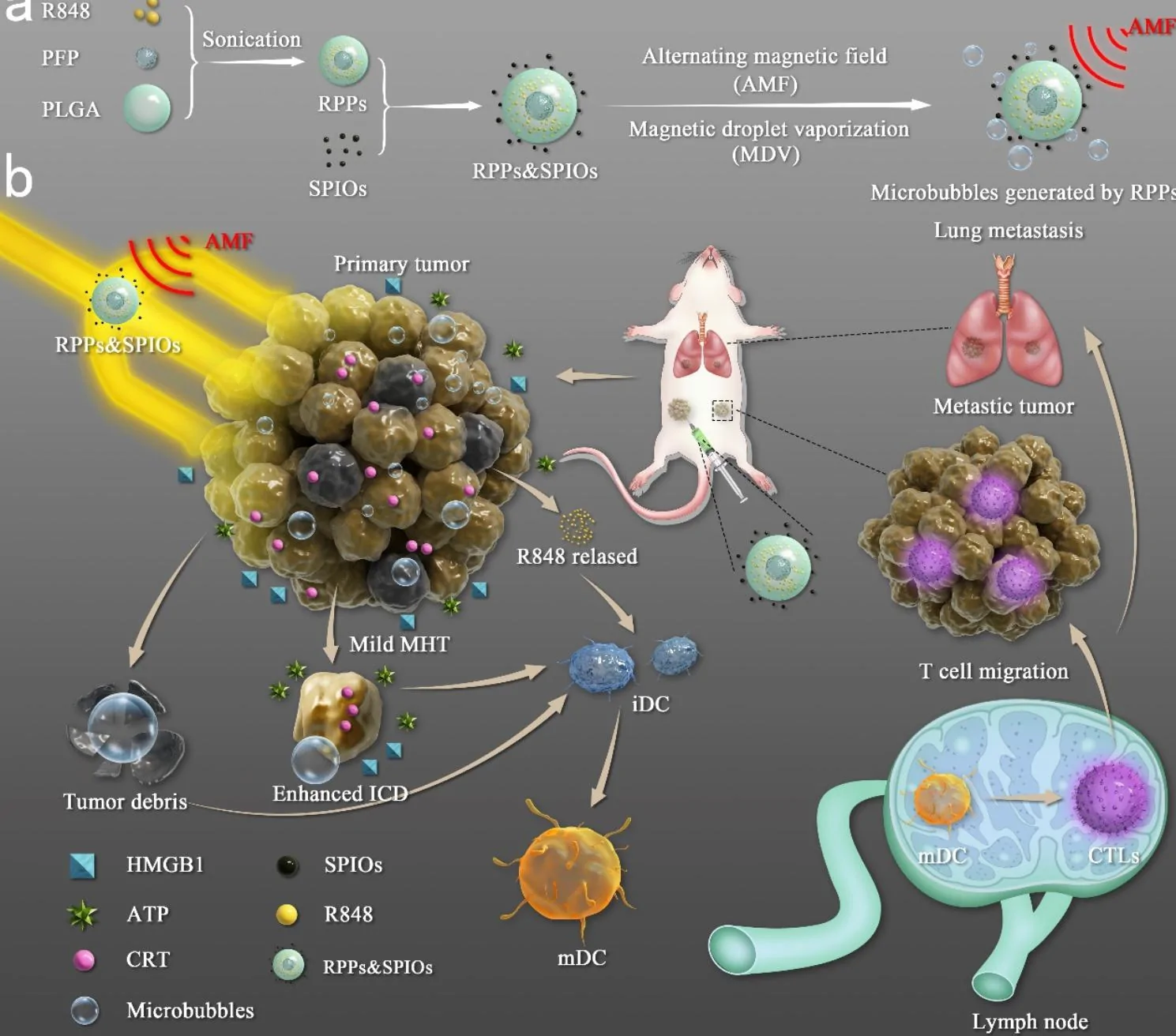
Schematic of the fabrication of SPIO + RPPs and the mechanism by which RPPs potentiate mild MHT against tumour proliferation and metastasis. **a**) Illustration of the synthesis of SPIO + RPPs and the process of MDV. **b**) The proposed mechanism of mild MHT potentiated by RPPs to prevent tumour proliferation and metastasis

## Results and discussion

### Characterization of RPPs and SPIOs

The obtained SPIOs were water-soluble with no aggregation but could be quickly aggregated by an external magnetic field (inserts in Fig. 1a), which suggested that the SPIOs can simultaneously achieve good suspension and fast magnetic response. Transmission electron microscopy (TEM) images showed that the obtained SPIOs were well dispersed (Fig. 1a) with a uniform size distribution of 11.93 ± 1.43 nm (Fig. 1b). As shown in Figure S1, the average hydrodynamic diameter of RPPs determined by dynamic light scattering (DLS) measurement was 37.48 ± 1.21 nm. From the hysteresis loops, the saturation magnetization of SPIOs and SPIOs + RPPs were 48.81 emu/g and 38.13 emu/g (Fig. 1c). Then, the magnetic-to-thermal conversion efficiency of SPIOs was evaluated under an alternating magnetic field (AMF). Thermal imaging showed that the magnetic hyperthermia effect of SPIOs in PBS increased rapidly with increasing concentrations of SPIOs (Fig. 1d, e). This excellent magnetic-thermal conversion efficiency could easily suffice for subsequent MHT. And we also detected the magnetothermal effect of the nanosystem (Figure S2a, b), indicating that the addition of RPPs would not affect the magnetic-to-thermal conversion efficiency of SPIOs. TEM imaging revealed that the RPPs possessed a smooth and uniform spherical morphology (Fig. 1f). The average hydrodynamic diameter of the RPPs determined by dynamic light scattering (DLS) measurement was 216.17 ± 2.12 nm (Fig. 1g). The average zeta potential of RPPs was − 8.47 ± 0.22 mV, which was suitable for applications in the biological milieu (Fig. 1h). The PFP-encapsulated nanodroplets were transformed into numerous microbubbles within 2 min when subjected to thermal stimulation and were visualized by optical microscopy (Fig. 1i). Microbubbles produced by phase-transition nanodroplets may cause mechanical damage to surrounding cells and lay the foundation for the potential to induce enhanced ICD. The UV − vis absorption spectra of RPPs and R848@PLGA(RPs) showed the characteristic absorption peak of R848 (324 nm), indicating the effective encapsulation of R848 into the nanodroplets (Figure S3a). According to the standard curves of R848 (Figure S3b), the loading efficiency and encapsulation efficiency of R848 were calculated to be 50.90% and 2.04%, respectively (Figure S3c). To investigate the release profiles of R848 from RPPs, RPs not containing PFP were also used as a control. With AMF exposure, the release of RPPs approached 72.44%, while RPs released only 49.09% of R848 (Fig. 1j), suggesting that under magnetic-thermal stimulation, the phase-transition effect could accelerate the release of R848.

### The enhanced ICD *in vitro* mediated by RPPs potentiated mild MHT

Before being applied in biological experiments, the biocompatibility of the hybrid nanosystem was evaluated in 4T1 cells. As shown in Figure S4a, b, c, there was no significant cytotoxicity to the cells after coincubation with SPIOs, RPPs and SPIOs + RPPs even up to 1 mg/mL, indicating their outstanding biocompatibility.

**Fig. 2:**
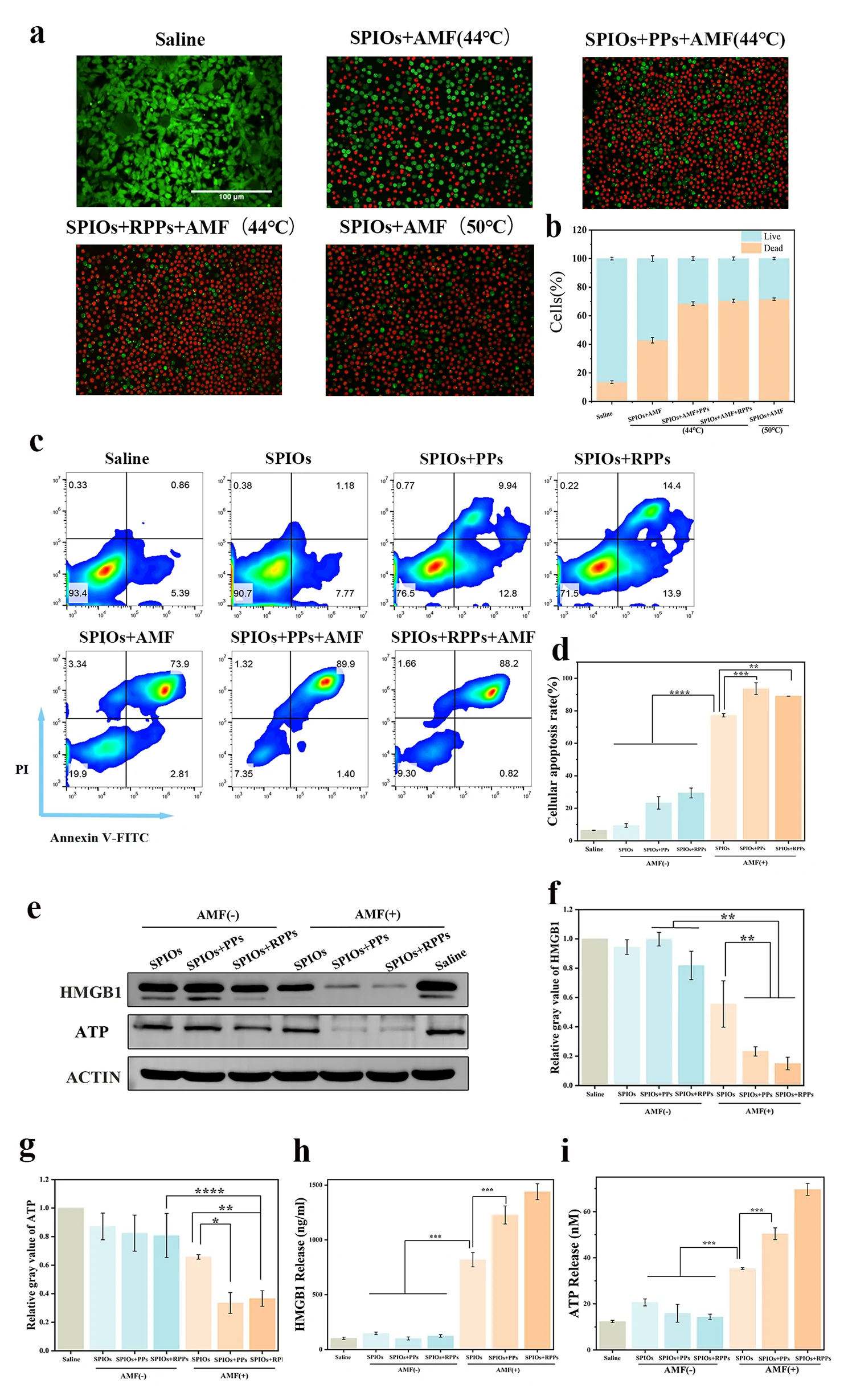
The enhanced ICD *in vitro* mediated by RPPs potentiated mild MHT. **a**) LSCM images of calcein-AM/PI-stained 4T1 cells under different treatment conditions. **b**) Quantitative analysis of (**a**). **c, d**) Flow cytometry analysis of cell apoptosis after different therapies (n = 3). **e**) Western blot analysis of HMGB1 and ATP expression in cells after various treatments. **f, g**) Quantitative levels of HMGB1 and ATP in 4T1 tumours after various treatments determined by Western blot analysis. (n = 3) **h, i**) ELISA kit analysis of the released HMGB1 and ATP in the cell supernatant. (one-way ANOVA with Tukey’s post hoc test, *P < 0.05, **P < 0.01, ***P < 0.001 and ****p < 0.0001)

First, the synergistic effect of liquid‒gas phase-transition nanodroplets in mild MHT was evaluated on 4T1 cells through confocal laser scanning microscopy (CLSM), where the live cells were stained by calcein-AM (green) and dead cells were stained by propidium iodide (PI, red). Five groups were set as follows: Saline, SPIOs + AMF (44℃) represented as mild MHT alone, SPIOs + AMF (50℃) represented as traditional MHT at high temperatures, SPIOs + PPs + AMF (44℃) and SPIOs + RPPs + AMF (44℃) represented as the combination of mild MHT and phase-transition nanodroplets–PFP@PLGA(PPs) or RPPs. As shown in Fig. 2a, b, the cellular apoptosis rate of mild MHT alone was only 42.91%, while traditional MHT at high temperatures resulted in a more significant apoptotic cell ratio (71.63%), but it was interesting that the cellular apoptosis rate reached 68.42% and 70.50% respectively when mild MHT combined with PPs or RPPs, comparing favourably with the cellular apoptosis rate of traditional MHT at high temperatures. It suggested that the deficit of mild MHT alone could be complement by the phase-transition nanodroplets, which could impart mechanical damage to cells associated with microbubbles expansion and implosion (cavitation effect) [21, 54].

Then, the therapeutic efficacy of RPPs in potentiating mild MHT was investigated in 4T1 cells *in vitro*. From the results of the CCK-8 assay (Figure S5), the groups receiving SPIOs, SPIOs + PP, and SPIOs + RPP exhibited unobvious cellular inhibition without AMF irradiation. Upon the application of AMF, the cellular inhibition rate was markedly enhanced. Additionally, with the utilization of PFP-containing phase-transition nanodroplets–PPs and RPPs, the groups treated with SPIOs + PPs + AMF and SPIOs + RPPs + AMF had a higher cellular inhibition rate (67.74% and 76.69%, respectively) than the group treated with SPIOs + AMF (47.45%). The result of the CCK-8 assay was further verified by flow cytometry apoptosis assay based on the Annexin V, FITC Apoptosis Detection Kit. As shown in Fig. 2c, d, the cellular apoptosis rate of the SPIO + PP + AMF group and SPIOs + RPPs + AMF group exceeded 90%, which was higher than that of SPIOs + AMF group (~ 77%), while 4T1 cells treated with SPIO, SPIO + PPs, or SPIO + RPPs without AMF were inhibited insignificantly.

Released HMGB1 and ATP are considered two representative markers of DAMPs, which serve as a “find me” signal for recruiting antigen presenting cells (APCs) [55]. Here, western blot analysis was used to detect the intracellular levels of HMGB1 and ATP. As shown in Fig. 2e, the protein bands clearly showed decreases in intracellular HMGB1 and ATP when mild MHT was applied. Among the three groups with AMF stimulation, the expression of HMGB1 and ATP inside cells of the SPIOs + AMF group was significantly higher than that of the groups of SPIOs + PPs + AMF or SPIOs + RPPs + AMF. The difference in the protein expression of these seven groups indicated that mild MHT and phase-transition nanodroplets both contributed to the release of HMGB1 and ATP. Three independent protein expression experiments calculated using the grey value on each strip of actin provided more accurate proof of this result (Fig. 2f, g). Furthermore, to validate the result in reverse, we detected the extracellular levels of HMGB1 and ATP in conditioned medium through enzyme-linked immunosorbent assay (ELISA) kit analysis (Fig. 2h, i). The results also suggested that mild MHT cooperating with phase-transition nanodroplets could promote the release of HMGB1 and ATP.

The above results revealed that the positive efficiency of phase-transition nanodroplets potentiated mild MHT, which had a stronger effect of inducing ICD than mild MHT alone, accompanied by more HMGB1 and ATP release.

### The effective activation of DCs *in vitro* mediated by RPPs potentiated mild MHT

**Fig. 3:**
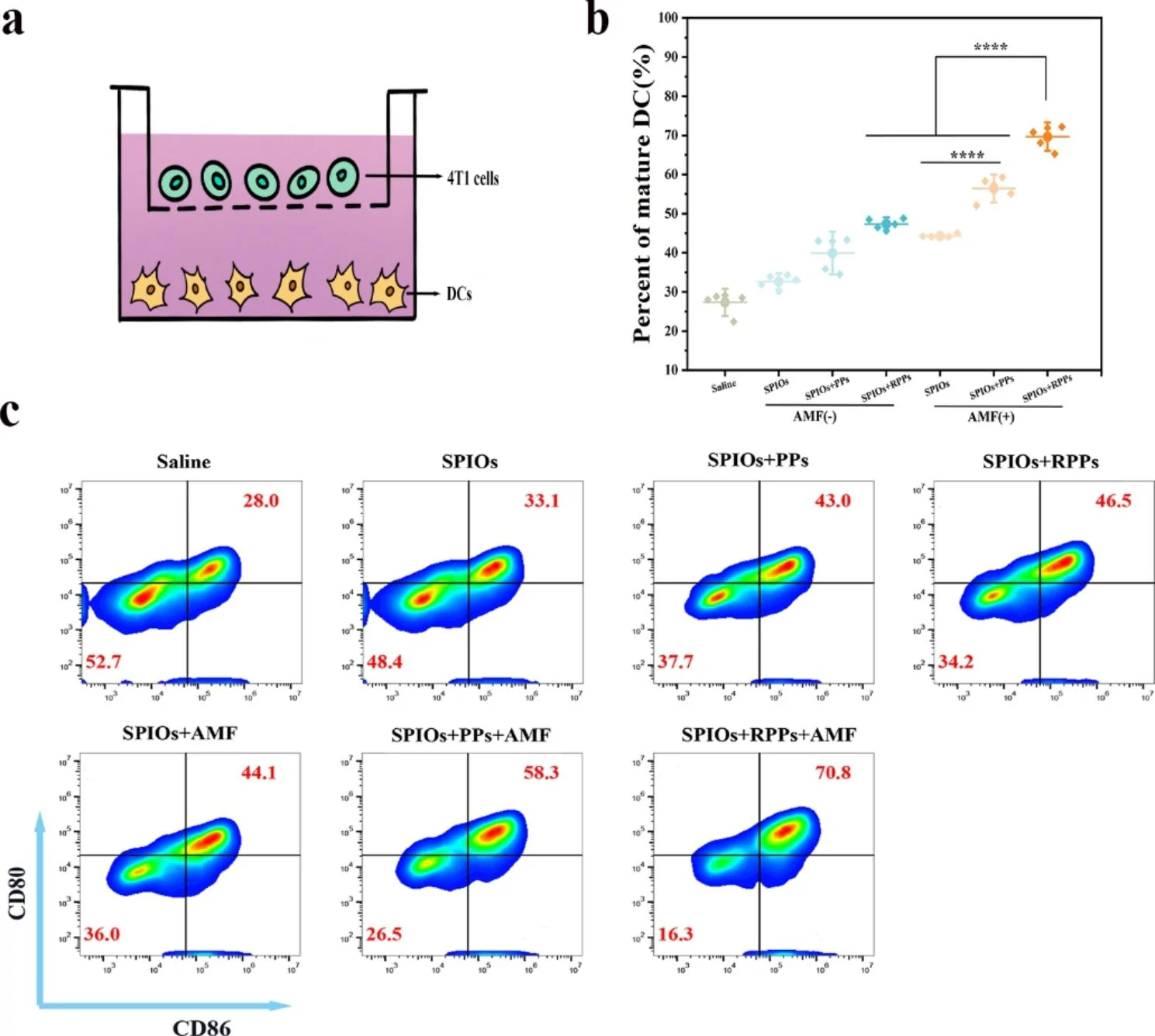
The activation of DCs *in vitro* mediated by RPPs potentiated mild MHT. **a**) Schematic illustration of the Transwell coculture system. **b, c**) The percentage of mature BMDCs after incubation with saline, SPIOs, SPIOs + PPs and SPIOs + RPPs for 12 h. In another set of experiments, DCs were cocultured with 4T1 breast tumour cells or with residues of 4T1 cells after MHT induced by SPIOs, SPIOs + PPs and SPIOs + RRPs for 12 h using Transwell coculture systems (n = 5). (one-way ANOVA with Tukey’s post hoc test, *P < 0.05, **P < 0.01, ***P < 0.001 and ****p < 0.0001)

The maturation of DCs, as the most powerful type of APC, is the essential initiator of T cell-mediated adaptive immunity and can be stimulated by antigens or immune adjuvants [56]. To further study the immune-activation abilities of SPIOs + RPPs, a coculture system of tumour cells and bone marrow-derived dendritic cells (BMDCs) (isolated from BALB/c mice) (Fig. 3a and S6) was constructed to investigate the immune stimulation effect of the hybrid nanosystem. The results of flow cytometry analysis (Fig. 3b, c) showed that the percentage of mature DCs (CD11 + CD80 + CD86+) significantly increased to 56.42% in the SPIOs + PPs + AMF group, while the DC maturation percentage was 44.32% in the SPIOs + AMF group. The elevated portion may be associated with the enhanced exposition of DAMPs induced by the destructive effect of microbubbles. Remarkably, the residues of 4T1 breast tumour cells after treatment with SPIOs + RPPs induced mild MHT showed dramatically enhanced DC maturation (69.66%), reaching a level much higher than that achieved by simply adding SPIOs + RPPs nanoparticles (47.34%) or coculturing with cell residues treated by SPIOs + AMF (44.32%) or SPIOs + PPs + AMF (56.42%). The data proved that the combination of mild MHT and RPPs could maximize the cancer vaccine-like function to greatly promote the maturation of DCs.

All of the above results suggested that the synergistic treatment strategy of utilizing RPPs to potentiate mild MHT plays an excellent role in boosting antigen release or exposure to elicit a stronger immunological response.

### The enhanced ICD ***in vivo*** mediated by RPPs potentiated mild MHT

First, the synergistic effect of RPPs in mild MHT was evaluated in 4T1 tumour-bearing BALB/c mice. From the H&E tumour slices under different treatment conditions (Fig S7, upper row) (grouping information was the same as in the *in vitro* experiment in Fig. 2a, b), the mild MHT alone showed cell volume reduction and nucleus shrinkage, while in the groups of the combination of mild MHT and phase-transition nanodroplets (SPIOs + PPs + AMF (44℃) and SPIOs + RPPs + AMF (44℃)), the H&E slices demonstrated severe damage, including the destruction of cell morphology, the rupture of cell nucleus, and apparent intercellular gaps as well as cavity structures, which may be caused by the mechanical forces from cavitation effect during MDV (such as cavitation-associated shear stress, shock waves, or microjets [27, 57]). In addition, to further verify the synergistic effect of RPPs in mild MHT, an immunofluorescence assay (IFA) of dissected tumours was also performed. From the TUNEL staining images, the mild MHT alone demonstrated no obvious cell apoptosis, while massive apoptotic cells could be observed after the combination of mild MHT with PPs or RPPs (Figure S7, middle row). The Ki67 staining images indicated that compared with mild MHT alone, the proliferation of tumour cells was more significantly inhibited after treatment with mild MHT combined with PPs or RPPs (Figure S7, lower row). The above results suggested that in the presence of RPs and PPs, the temperature of MHT could be reduced to approximately 44 °C to achieve a comparable therapeutic effect to that of traditional high-temperature MHT at 50 °C, which was consistent with the *in vitro* experimental results.

**Fig. 4:**
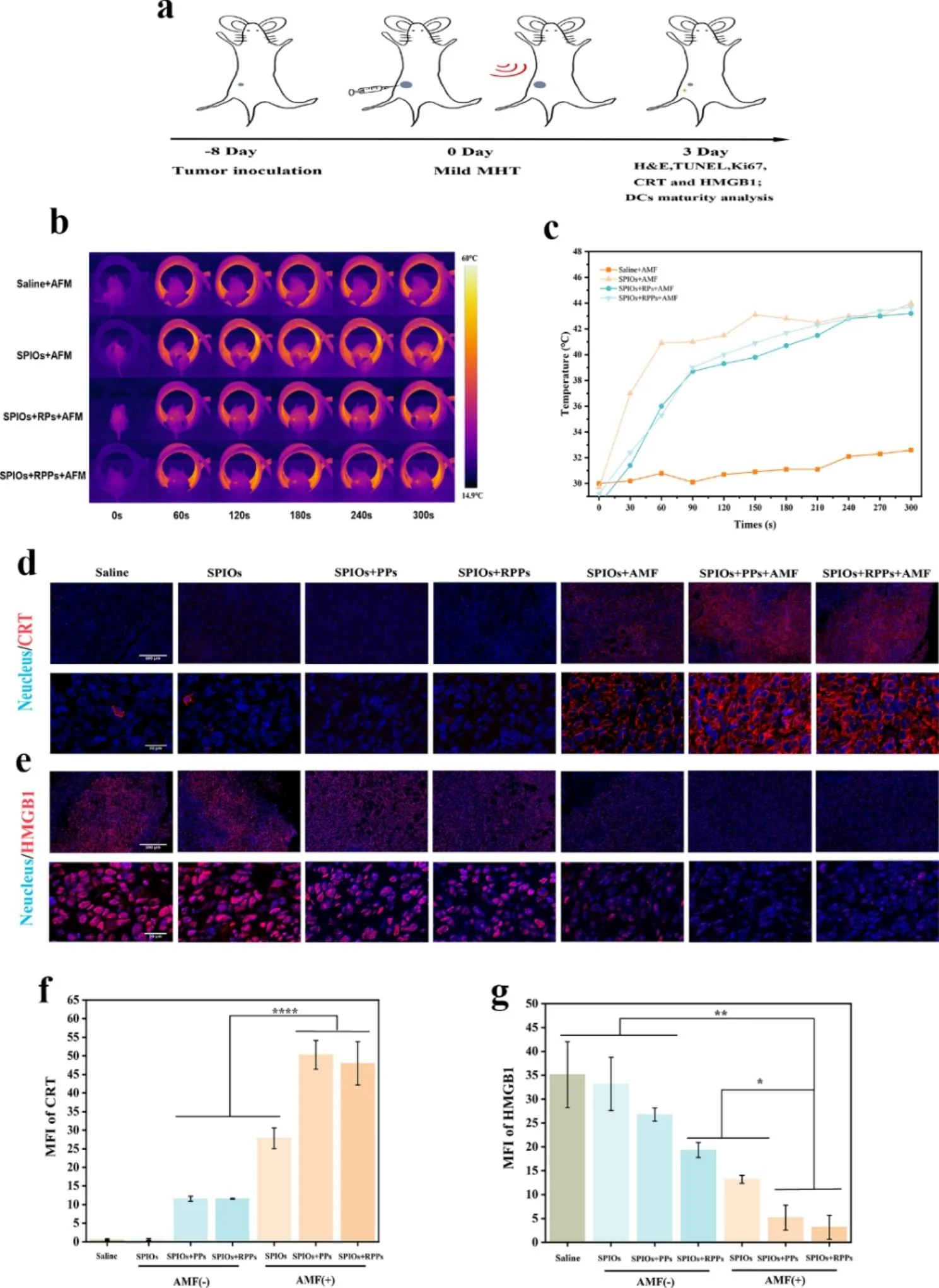
The enhanced ICD *in vivo* mediated by RPPs potentiated mild MHT. **a**) Schematic illustration of the *in vivo* experimental design. **b**) IR thermal images of 4T1-tumour-bearing mice undergoing mild MHT. **c**) Quantitative temperature rise curves based on IR thermal imaging data in a. **d, e**) Immunofluorescence staining images of HMGB1/CRT in tumour tissue after the different treatment strategies. The upper panel is under low magnification (scale bars = 200 μm), and the lower panel is under high magnification (scale bars = 20 μm). **f**) Mean fluorescence intensity (MFI) of CRT (n = 3). **g**) Mean fluorescence intensity (MFI) of HMGB1 in nuclear

In subsequent *in vivo* experiments, 4T1 tumour-bearing BALB/c mice were randomly divided into 7 groups (n = 5) to receive saline, SPIOs, SPIOs + PPs, and SPIOs + RPPs with and without AMF stimulation (Fig. 4a). Given the application scenario of SPIOs + RPPs where the treatment and visual monitoring of primary tumour could be performed simultaneously based on the defined diagnosis and localization of tumour and the trade-off between therapeutic safety and effectiveness,

intratumoral administration with a small dose was a more safe and reasonable choice in such application scenario, while intravenous injection with same dose could be insufficient for the tumour accumulation and therapeutic effect. As shown in Fig. 4b, the temperature of the subcutaneous tumour reached approximately 43 °C under AMF after intratumoral injection of SPIOs, SPIOs + PPs, and SPIOs + RPPs and remained between 43 and 44 °C for 10 min (Fig. 4c), which signifies that mild MHT could be readily realized *in vivo*. During treatment, the growth behaviours of tumours in all mice were recorded. Figure S8 a-c indicates that the delay of 4T1 tumour progression suffered great failure in the saline, SPIOs, SPIOs + PPs, and SPIOs + RPPs groups, while mild MHT alone and mild MHT potentiated by PPs and RPPs significantly inhibited tumour growth. Remarkably, among the three groups with AMF stimulation, the combination of mild MHT and RPPs achieved the most obvious tumour inhibition effect, even providing opportunities to eliminate tumours completely.

We also verified the levels of DAMPs in tumours after different treatments *in vivo*. CRT is exposed on the cell membrane surface, which serves as an “eat me” signal to promote phagocytosis by APCs [58]. According to the CRT staining images and corresponding semiquantitative analysis, the application of AMF significantly promoted the exposure of CRT compared with the no AMF stimulation groups. The exposure of CRT increased by 80.60% and 72.39% in the SPIO + PPs + AMF and SPIO + RPPs + AMF groups, respectively, compared with that in the group receiving mild MHT alone (Fig. 4d, f). HMGB1, as a nucleus-binding protein, is overexpressed in tumours. As shown in Fig. 4e, g, under AMF stimulation, the content of HMGB1 decreased apparently, while the groups without AMF stimulation showed a higher content of HMGB1 in tumour tissue. The release of HMGB1 of tumour tissue increased by 36.55% and 45.84% in the groups receiving SPIOs + PPs + AMF or SPIOs + RPPs + AMF, respectively, compared with the SPIOs + AMF group. The analysis of CRT exposure and HMGB1 release of tumour tissues has proven that RPPs potentiated mild MHT and could boost the impact of ICD *in vivo* at a comparatively comfortable and safe temperature. The increased immunogenicity of the TME paved the way for the subsequent immune response activation.

### The potent activation of DCs *in vivo* mediated by RPPs potentiated mild MHT

**Fig. 5:**
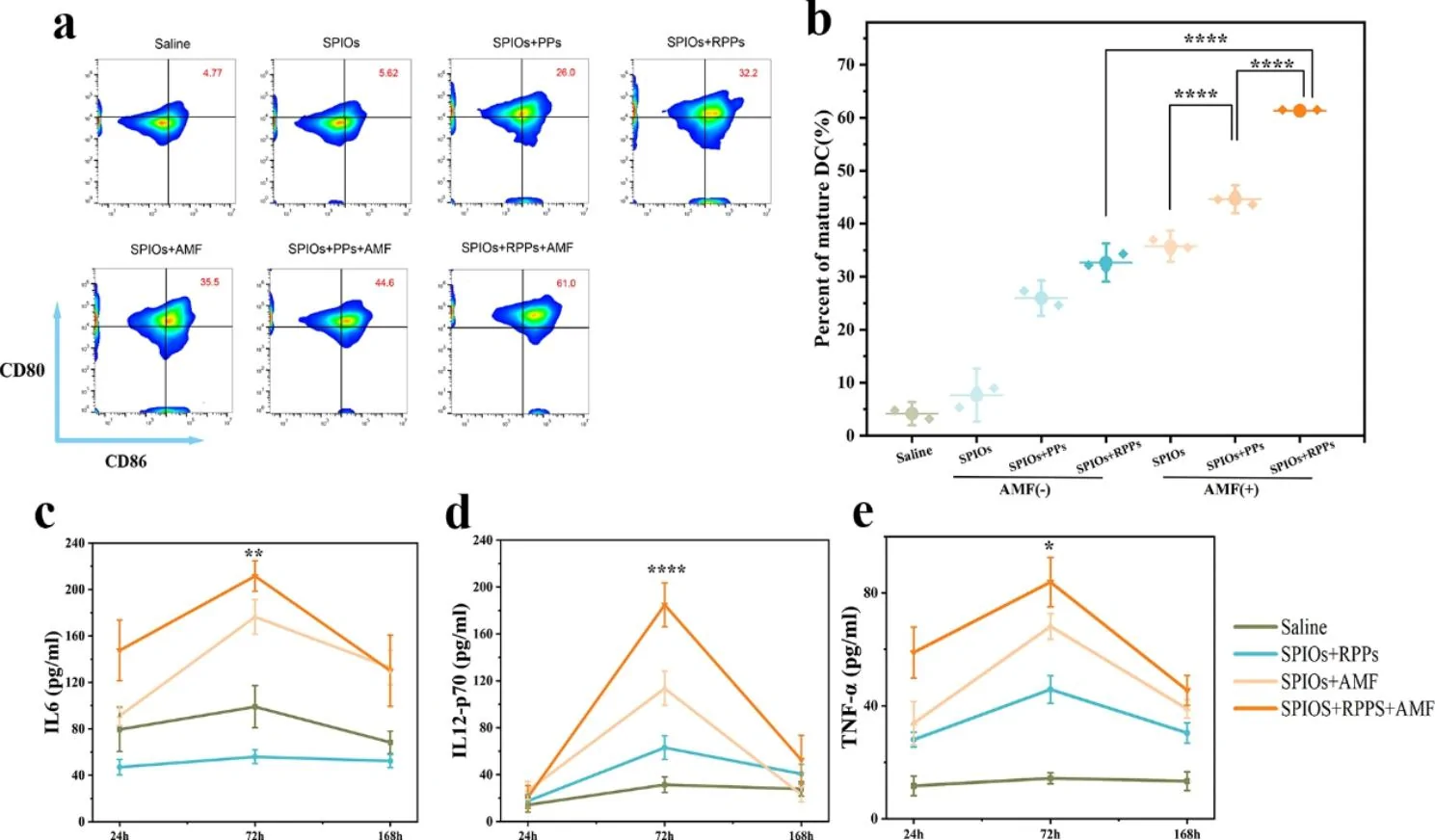
The potent activation of DCs *in vitro* mediated by RPPs potentiated mild MHT. **a**) Flow cytometric analysis of DC maturation in tumour-draining lymph nodes of mice in different treatment groups. **b**) The corresponding quantification of DC maturation (n = 5). **c, d, e**) Serum levels of IL-6, TNF-α, and IL-12 in mice isolated at 24 h, 72 and 168 h after different treatments. For c-e, P values were determined between the third group (SPIOs + AMF) and the fourth group (SPIOs + RPPs + AMF) (one-way ANOVA with Tukey’s post hoc test, *P < 0.05, **P < 0.01, ***P < 0.001 and ****p < 0.0001)

In further *in vivo* experiments, the DC status was analysed accordingly. Flow cytometry analysis of draining lymph nodes (Fig. 5a, b) showed that compared with the SPIOs + AMF group (35.73%), the percentage of mature DCs of the SPIO + PPs + AMF group (44.63%) showed a slight increase of approximately 8.9%, which was consistent with the *in vitro* results. Remarkably, the SPIOs + RPPs + AMF group induced significant DC maturation (61.33%), which appeared to be much higher than that observed in the SPIOs + RPPs group without AMF stimulation (32.67%) or SPIOs + PPs + AMF in the absence of R848 (44.63%). Mild MHT combined with phase-transition nanodroplets facilitated the exposure of TAAs, including DAMPs, which could effectively recruit DCs and stimulate the maturation of DCs, particularly with the help of the immune adjuvant.

Furthermore, the blood of mice was collected at different periods after different treatments to monitor potential changes in cytokines and proinflammatory mediators, including IL-6, IL-12, and TNF-α, using ELISA. Compared with the SPIOs + PPs + AMF and SPIOs + AMF groups, the SPIOs + RPPs + AMF group showed sustainable promotion of the secretion of IL-6, IL-12, and TNF-α in the observation period (Fig. 5c, d, e), which verified that mild MHT could efficiently promote the maturation of DCs, especially in the presence of RPPs.

Therefore, the SPIOs + RPPs system has the outstanding ability to induce enhanced ICD and strengthen the immunogenicity of the TME, thus providing a robust vaccine-like effect on the activation of DCs.

### The significant inhibition of tumour growth and metastasis mediated by RPPs potentiated mild MHT

**Fig. 6:**
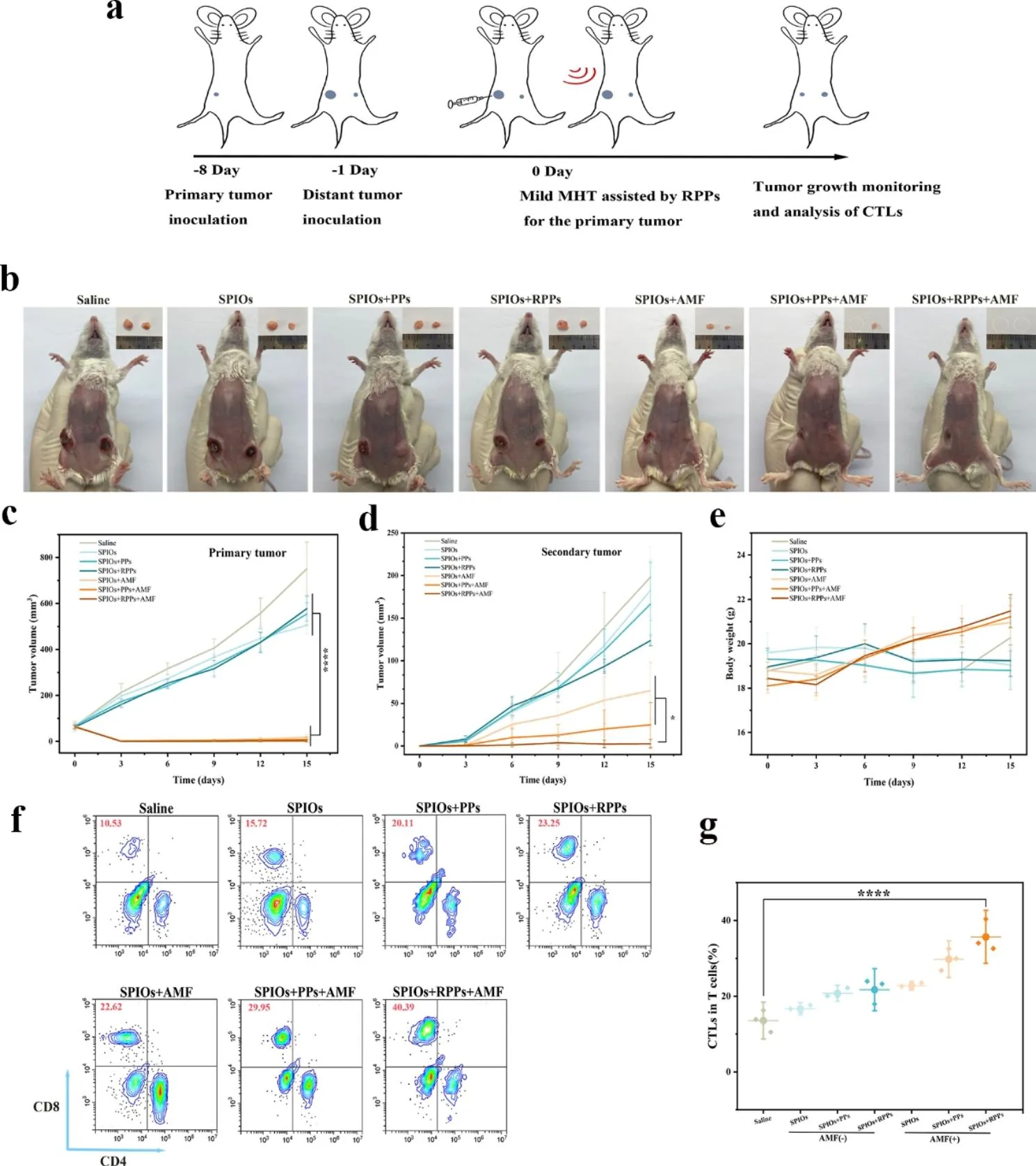
The significant inhibition of tumour growth and metastasis mediated by RPPs potentiated mild MHT. **a**) Schematic illustration of the *in vivo* experimental design. **b**) Digital photos of 4T1 tumours on both sides *in vivo* and ex vivo on 15 day 27 after different treatments. **c, d**) Growth curves of the primary tumours and the distant tumours in different groups (n = 5). **e**) Analysis of body weights of mice in different treatment groups. **f**) Representative flow cytometric analysis of CTLs in the distant metastatic tumours of mice in different groups. g) Quantification of CTLs (n = 5, one-way ANOVA with Tukey’s post hoc test, ****p < 0.0001)

To interrogate the robust immunological responses induced by the synergy of mild MHT and RPPs, a bilateral orthotopic 4T1 tumour model was established to mimic distant metastasis, and the growth of the primary tumour and distant tumours were evaluated in detail. The design of our animal experiment is shown in Fig. 6a. The development of primary tumours and distant tumours in these groups was measured every three days (Fig. 6b, c, d). The results demonstrated that without AMF stimulation, SPIOs, SPIOS + PPs, or SPIOs + RPPs could not suppress either the primary tumour or metastatic tumour. As another control, mild MHT alone could inhibit the growth of primary tumours significantly, but the growth speed of metastatic tumours could only be slightly delayed, whereas it was further slowed by mild MHT combined with PPs. Interestingly, RPPs potentiated mild MHT, not only providing opportunities to eliminate the primary tumours completely but also showing almost complete inhibition of the metastatic tumour. Moreover, there was no obvious body weight loss throughout the treatment process (Fig. 6e). For SPIOs + RPPs, no significant pathological changes were found in major organs (heart, liver, spleen, lung, and kidney) in the histological examination of mice (Figure S9a, b), and no abnormal indexes were observed in the blood biochemical and routine blood test of mice, demonstrating the high safety of SPIOs + RPPs.

To examine the immune cascade induced by the treatment, metastatic tumours were harvested for flow cytometry analysis to evaluate the CTL response *in vivo* (Fig. 6f, g). The SPIO + RPPs + AMF group displayed the highest rate of CTLs (CD8+) infiltration in tumours, 35.67% of total tumour cells, which was ~ 26% more than that of the saline group and ~ 17% more than that of the SPIOs + AMF group. As shown in Figure S10, obvious fluorescence signals (red) of the specific surface antigen CD8 (CD8+) expressed by T cells were observed in the SPIOs + RPPs + AMF group, indicating that the antitumour immune response was enhanced, leading to the infiltration of CTLs into the tumour site. These results proposed that the efficacy of mild MHT could be greatly potentiated by RPPs to inhibit both primary tumours and distant tumours.

**Fig. 7:**
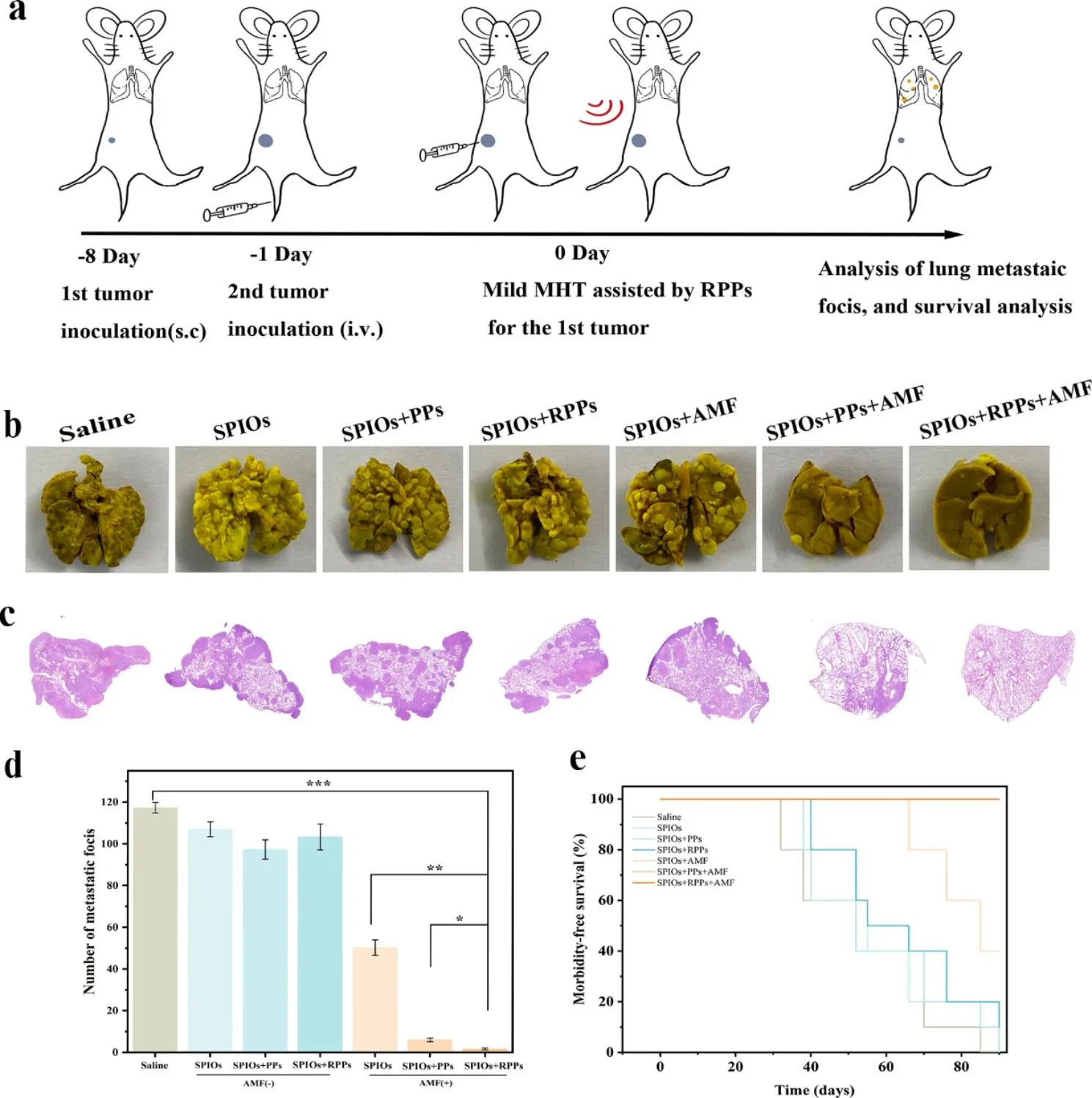
The remarkable inhibition of lung metastasis mediated by RPPs potentiated mild MHT. **a**) Schematic illustration of the *in vivo* experimental design. **b**) Photographs of lung metastatic nodules from each group. **c**) H&E staining images (1×) of lung metastasis. **d**) Quantification of pulmonary metastasis nodes in mice after different treatments. **e**) Morbidity survival analysis of mice in each group. (n = 5, t test, *p < 0.05, **p < 0.01, ***p < 0.001)

After 28 days of treatment (Fig. 7a), the mice in the different groups were sacrificed to study the extent of metastasis to the lung. As shown in Fig. 7b, d, the white-light images showed that there were more than 100 tumour nodules in the lung in the groups treated with saline, SPIOs, SPIOs + PPs, SPIOs + RPPs groups, while the number of tumour nodules was reduced by more than half in the mice receiving mild MHT alone. More interestingly, after mild MHT combined with PPs and RPPs, the number of metastatic foci in the lungs of mice was severely reduced to ~ 6 and ~ 2, respectively, which was also observed by HE staining (Fig. 7c). The data indicated that the synergistic mild magnetic hyperthermia immunotherapy activated by SPIOs + RPPs could promisingly suppress tumour metastasis. Additionally, the SPIOs + RPPs + AMF group achieved a 100% survival rate of mice at 90 days (Fig. 7e), demonstrating that mild MHT assisted by RPPs could effectively delay the recurrence or metastasis of tumours, achieving a good prognosis.

We presumed that the enhanced effect of inhibiting metastasis in the SPIOs + PPs + AMF group may be attributed to the highly immunogenic TME created by the combination of mild MHT and cavitation effect of microbubbles produced by phase transition nanodroplets, which evoked a more intense immune response than mild MHT alone. In addition, the utilization of immune adjuvant advanced in the amplification of immunogenicity in the TME further, which helped to reverse the immunosuppressive TME, sensitize tumours to immunotherapy, and provoke host immunity, enabling the SPIOs + RPPs + AMF group to achieve such an optimal effect in suppressing the recurrence and metastasis of tumours.

### *In vitro* and *in vivo* magnetic field-responsive ultrasound imaging

**Fig. 8:**
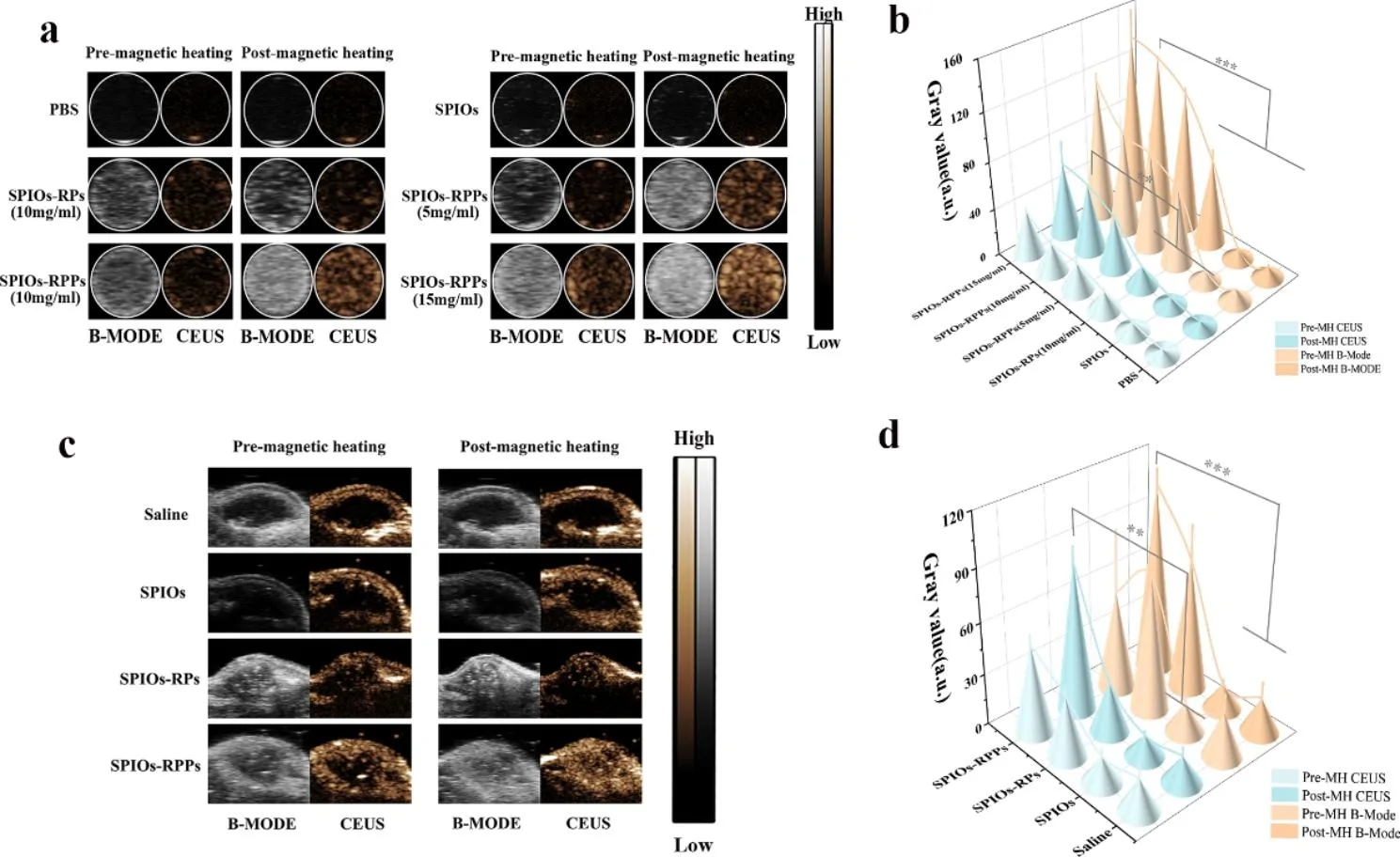
The remarkable inhibition of lung metastasis mediated by RPPs potentiated mild MHT in both *in vitro* and *in vivo* magnetic field-responsive ultrasound imaging. **a**) *In vitro* ultrasound imaging of saline, SPIOs, SPIOs + RPs, and SPIOs + RPPs at different RPP concentrations in B-mode and CEUS mode before and after magnetic heating. **b**) Quantitative grey values of ultrasound images corresponding to images of (**a**) (n = 3). **c**) Ultrasound imaging of mice bearing 4T1 tumours with saline, SPIOs, SPIOs + RPs, and SPIOs + RPPs in B-mode and CEUS mode before and after magnetic heating. **d**) Quantitative grey values of ultrasound images corresponding to images of (**c**). (n = 5, one-way ANOVA with Tukey’s post hoc test, **p < 0.01, ***p < 0.001)

Microbubbles are also generally used as ultrasound contrast agents in the clinic [59]. Currently, there are two phase-transitional pathways named after their activation methods. Acoustic droplet vaporization (ADV) is activated by ultrasound, and optical droplet vaporization (ODV) is triggered by laser [60]. However, ADV is vulnerable to skeletal or gas interference with sonic propagation, and ODV is always limited to the optical penetration depth [61, 62]. To solve the critical limitations of ADV and ODV and to realize highly efficient MHT with real-time guidance capability, our previous research proposed a new liquid–gas phase-transformation strategy, that is, magnetic droplet vaporization (MDV), for efficient magnetic field-responsive cancer theranostics [29]. In this work, the MDV of the hybrid nanosystem utilized the thermal energy of mild MHT to subsequently induce the vaporization of RPPs. Systematic *in vitro* and *in vivo* investigations have been conducted to demonstrate the feasibility and efficiency of MDV for cancer theranostics. The high magnetic-thermal transfer efficiency of the hybrid nanosystem was expected to facilitate the induction of MDV. The generation of microbubbles through MDV was observed in situ by *in vitro* ultrasound imaging (Fig. 8a). There were no obvious changes in ultrasound signals in the PBS and the suspension of SPIOs before and after exposure to AMF. Significantly improved contrast-enhanced ultrasonography was observed in suspensions of SPIOs + RPPs in B-mode and CEUS mode after magnetic heating. Moreover, the contrast enhancement of ultrasound imaging was also dependent on the RPP concentration, and the data showed that 10 mg/ml RPPs made it possible to obtain the optimal comparison before and after magnetic heating. A significant contrast enhancement in CEUS mode (Fig. 8b) was a powerful demonstration that a large number of microbubbles can be generated by the transition of RPPs after exposure to thermal stimulation because only contrast-based ultrasound imaging modality could respond to microbubbles.

The performance of ultrasound imaging *in vivo* with SPIOs + RPPs was further evaluated in BALB/c mice bearing 4T1 breast cancer. After the intratumoral administration of saline, SPIOs, SPIOs + RPs, or SPIOs + RPPs, the mice were exposed to the AMF for magnetic heating and a further MDV procedure. The images showed that only SPIOs + RPPs displayed outstanding ultrasound imaging capacity in both B-mode and CEUS mode, while there were no obvious ultrasound signal changes after exposure to AMF in the other groups (Fig. 8c). The quantitative grey values (Fig. 8d) further revealed that the ultrasound signal intensity increased by ~ 67%, whereas the other three groups had no obvious signal intensity elevation, demonstrating the high MDV efficiency *in vivo* of RPPs for ultrasound imaging.

The excellent ultrasound imaging capacity makes it possible to visualize the MHT process in tumour sites, providing guidance and monitoring for therapy.

## Conclusion

In summary, the utilization of RPPs could lower the effective temperature threshold of MHT from 50 ℃ to 44 ℃ through the cavitation effect, still keeping comparative tumour suppression effects. SPIOs + RPPs mediated mild MHT could induce enhanced ICD accompanied by increased release of HMGB1 and ATP from cells and greater exposure of CRT on the cell membrane compared with mild MHT alone. In addition to eliminating the primary tumour, SPIOs + RPPs mediated mild MHT promoted the maturation of DCs both *in vitro* and *in vivo*, thus activating CTLs to inhibit contralateral metastasis and lung metastasis. Moreover, the MDV ability of the hybrid nanosystem can help realize the excellent function of ultrasound imaging, providing the potential to guide or monitor the treatment process. Considering the good biocompatibility and high biosafety of SPIOs + RPPs, the proposed synergistic treatment technique may have bright prospects for future clinical translation, and we will carry out a preclinical study on the hybrid nanosystem-potentiated mild MHT. In summary, we provide a novel strategy for enhanced mild magnetic hyperthermia immunotherapy and ultrasound imaging with great clinical translation potential.

## Electronic supplementary material

Below is the link to the electronic supplementary material.

Supplementary Material 1: Hydrodynamic size distribution of RPPs by DLS (Figure S1); The magnetothermal effect of the nanosystem (Figure S2);UV − vis absorption spectra of RPPs and the encapsulation efficiency and loading efficiency of RPPs (Figure S3); Cell viabilities after incubation with SPIOs, RPPs and SPIOs + RPPs (Figure S4);CCK-8 assay of the cellular inhibition rate after different treatments (Figure S5); Morphology of BMDCs under optical microscope (Figure S6); Representative images of H&E staining, TUNEL, and Ki67 immunostaining of tumour slices after different treatments (Figure S7); The analysis of tumour growth after different treatments (Figure S8); Biosafety analysis of SPIOs + RPPs *in vivo* (Figure S9); CD8 + immunostaining of distant metastatic tumour slices after different treatments (Figure S10). (PDF)

## Data Availability

The datasets used and/or analysed during the current study are available from the corresponding author on reasonable request.
