# Correction: Phase-transition nanodroplets with immunomodulatory capabilities for potentiating mild magnetic hyperthermia to inhibit tumour proliferation and metastasis

**DOI:** 10.1186/s12951-026-05103-9

**Published:** 2026-10-07

**Authors:** Qiaoxi Qin, Yang Zhou, Pan Li, Ying Liu, Ruxi Deng, Rui Tang, Nianhong Wu, Li Wan, Ming Ye, Hong Zhou, Zhiming Wang

**Affiliations:** 1 Department of Ultrasound, Affiliated Hospital of Southwest Jiaotong University, The Third People’s Hospital of Chengdu, Chengdu, 610031 China; 2 Institute of Biomedical Engineering, College of Medicine, Southwest Jiaotong University, Chengdu, 610031 China; 3 Department of Ultrasound, The Second Affiliated Hospital of Chongqing Medical University, Chongqing, 400010 China; 4 Institute of Ultrasound Imaging of Chongqing Medical University, Chongqing, 400010 China; 5 Department of Health Management (Physical Examination) Center, The Second Affiliated Hospital of Chongqing Medical University, Chongqing, 400010 China

**Correction: Journal of Nanobiotechnology (2023) 21:131**

**https://doi.org/10.1186/s12951-023-01885-4**

In this article Fig. 2 appeared incorrect: image-placement errors occurred during the figure assembly process. The original image files were assigned relatively complex and similar numerical identifiers. This led to a misidentification of the image numbers when the representative images were selected and assembled into the figures.

Specifically, in Fig. 2a, another image (NO.3-2-20-1) from the SPIOs + PPs+AMF (44 °C) group was inadvertently placed in the panel labelled “SPIOs + AMF (50°C)”, while the intended representative image (NO.5-2-20-1) for the SPIOs + AMF (50 °C) group was omitted. The misplaced image and the image correctly shown for the SPIOs + PPs+AMF (44 °C) group were acquired separately, but their microscopic fields of view partially overlapped, which results in the shared cellular features observed between the two panels. Because the fluorescence microscopy images from the two experimental groups appeared visually similar, the error was not identified during the subsequent figure review and manuscript proofreading processes.

For completeness and transparency, the old incorrect and correct versions of Fig. 2 are displayed below.

Incorrect Fig. 2

**Figure:**
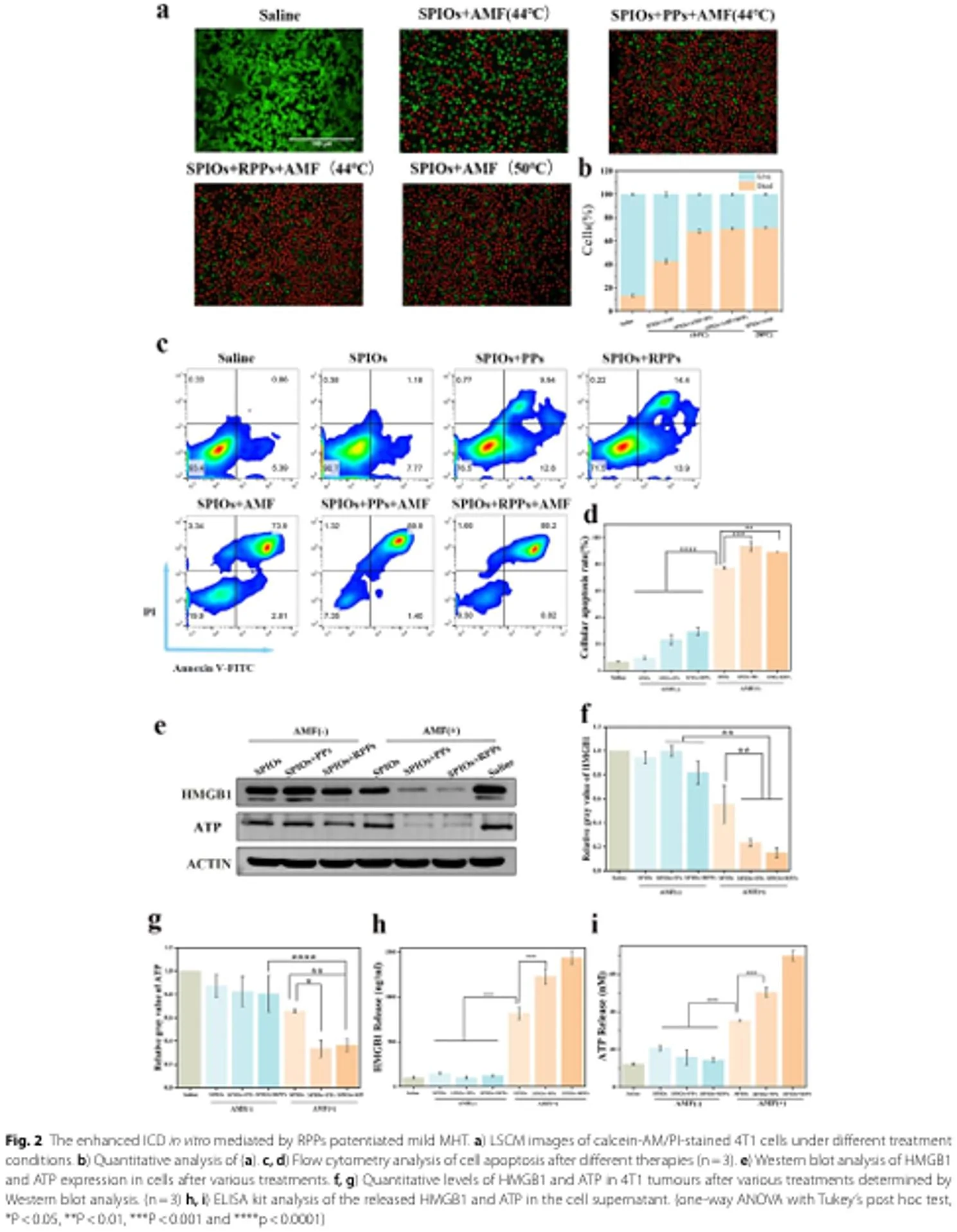

Correct Fig. 2.

**Fig. 2:**
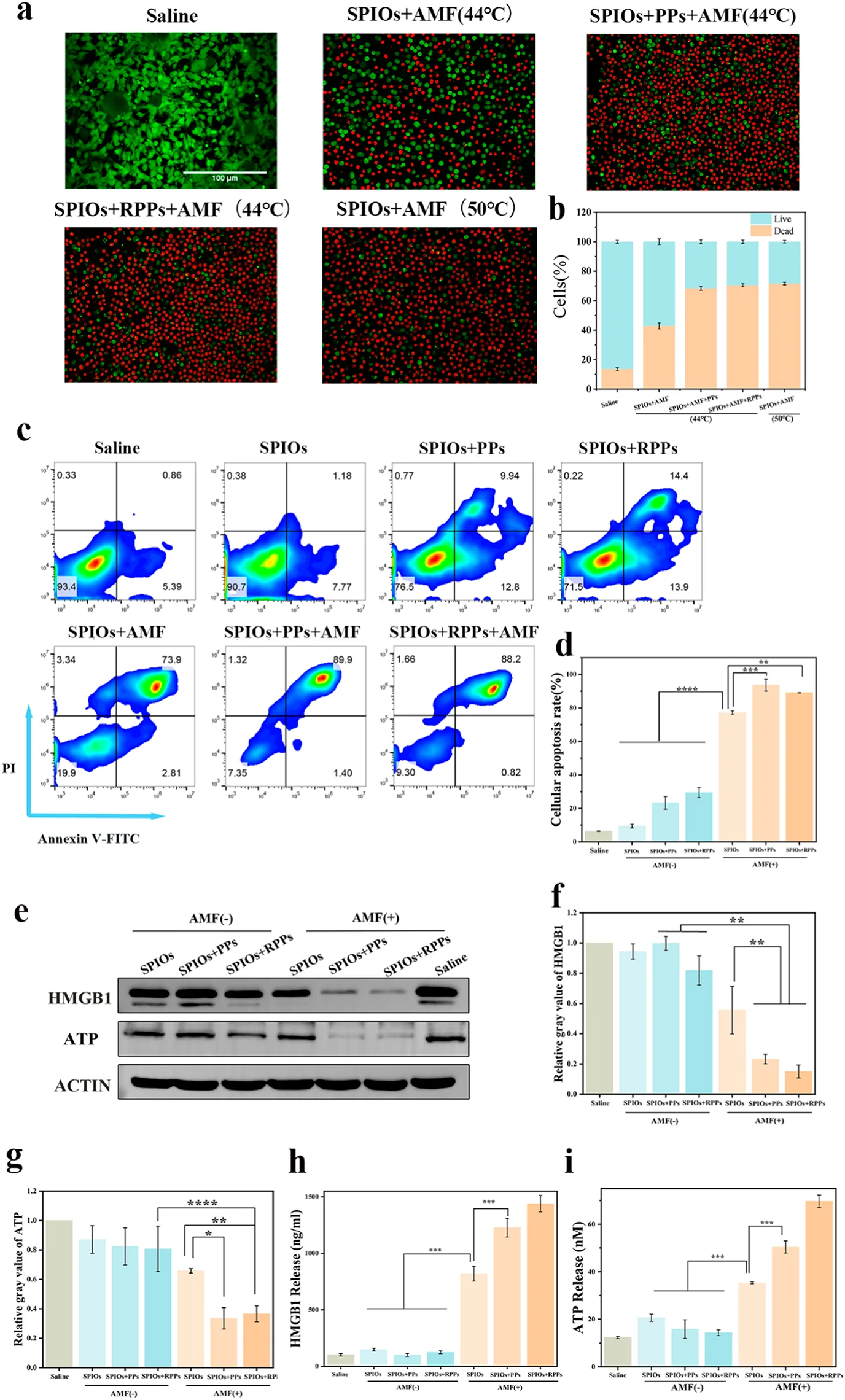
The enhanced ICD in vitro mediated by RPPs potentiated mild MHT. **a**) LSCM images of calcein-AM/PI-stained 4T1 cells under different treatment conditions. **b**) Quantitative analysis of (**a**). **c**,** d**) Flow cytometry analysis of cell apoptosis after different therapies (*n* = 3). **e**) Western blot analysis of HMGB1 and ATP expression in cells after various treatments. **f**,** g**) Quantitative levels of HMGB1 and ATP in 4T1 tumours after various treatments determined by Western blot analysis. (*n* = 3) **h**,** i**) ELISA kit analysis of the released HMGB1 and ATP in the cell supernatant. (one-way ANOVA with Tukey’s post hoc test, *P < 0.05, **P < 0.01, ***P < 0.001 and ****p < 0.0001)

The original article has been corrected.

