# Supplementary material for: Phase-transition nanodroplets with immunomodulatory capabilities for potentiating mild magnetic hyperthermia to inhibit tumour proliferation and metastasis
Source: J Nanobiotechnology. 2023 Apr 17;21:131. doi: 10.1186/s12951-023-01885-4 (PMC10108485; doi:10.1186/s12951-023-01885-4)
Supplement: Supplementary file 1 — Supplementary Material 1: Hydrodynamic size distribution of RPPs by DLS (Figure S1); The magnetothermal effect of the nanosystem (Figure S2);UV − vis absorption spectra of RPPs and the encapsulation efficiency and loading efficiency of RPPs (Figure S3); Cell viabilities after incubation with SPIOs, RPPs and SPIOs + RPPs (Figure S4);CCK-8 assay of the cellular inhibition rate after different treatments (Figure S5); Morphology of BMDCs under optical microscope (Figure S6); Representative images of H&E staining, TUNEL, and Ki67 immunostaining of tumour slices after different treatments (Figure S7); The analysis of tumour growth after different treatments (Figure S8); Biosafety analysis of SPIOs + RPPs in vivo (Figure S9); CD8 + immunostaining of distant metastatic tumour slices after different treatments (Figure S10). (PDF) [file 12951_2023_1885_MOESM1_ESM.docx]

Supplementary material

Phase Transition Nanodroplets with Immunomodulatory Capabilities for Potentiating Mild Magnetic Hyperthermia to Inhibit Tumor Proliferation and Metastasis

**Experimental Section**

**1.Materials**

Carboxy-terminated polylactic acid/glycolic acid PLGA (PLGA-COOH polymerization ratio: 50:50, molecular weight: 12,000 Da) was purchased from Jinan Daigang Biotechnology Co.,Ltd. (China). Polyvinyl alcohol (PVA) was obtained from Sigma-Aldrich (USA). R848 were purchased from Macklin (China). Liquid PFP was obtained from Alfa Aesar (UK). Calcein-AM, PI and CCK-8 assay kits were purchased from Dojindo Laboratories (Japan). ELISA kits were purchased from Dakewe biotech (China). Antibodies to cell surface markers for flow cytometry analysis were purchased from BioLegend, Inc (USA). Ferric chloride (FeCl_3_·6H2O), ferrous chloride (FeCl_2_·4H2O), and sodium hydroxide (NaOH) were purchased from Sigma-Aldrich (USA). All unspecified reagents used were of analytical grade or better.

**2.Methods**

**2.1 Synthesis of SPIOs+RPPs:**

**a) Synthesis of RPPs:** The liquid-gas phase-transition nanodroplets loading R848 were fabricated by using a typical double-emulsion process (w/o/w). [1, 2] Briefly, R848 was dissolved in DMSO at 10 mg ml^-1^.20ul R848 and 200μL PFP were added to 1 ml PLGA (25 mg ml^-1^) dissolved in dichloromethane. Then, the mixture was emulsified by using an ultrasonic probe (Sonics & Materials, Inc., USA) with power of 60 W for 3 min to form the first w/o emulsion. The w/o emulsion was then poured into 5mL 4% w/v cold PVA solution and emulsified for 3 min at the power of 45W for the second w/o/w emulsion. Subsequently, 10 mL 2% v/v of isopropyl alcohol solution was added to the resulting emulsion and stirred until chloroform volatilization. Lastly, the RPPs were collected after centrifugation at 10,000×g for 10 min. As for the preparation of RPs and PPs, the difference lies in the removal of PFP or R848.

**b) Synthesis of SPIOs:** First, SPIOs were prepared by co-precipitation method.[3] 2 g of FeCl_2_·4H2O and 5.2 g of FeCl_3_·6H2O was added to a three-necked flask containing 12.5 ml deionized water stirred for 10 min. Second, HCl (4.25 M, 0.85 ml) was added into this mixture solution to ensure the balance of Fe (II) and Fe (III) ions. Third, the three-necked flask was heated to 100 ℃ in a water bath. Fourth, NaOH solution (1.5 M, 250 ml) was added dropwise to the mixture solution rotating at 700 rpm and stirred for 1 h to precipitate. The precipitated SPIOs was collected by using a magnet. Finally, the precipitate was washed with deionized water until the pH of the suspension became neutral, and the precipitated SPIOs aqueous dispersion was lyophilized in vacuum at -80℃ to obtain the purified.

- 1. **Characterization of SPIOs+RPPs:**

1. **Characterization of RPPs:**

The morphology of the resulting RPPs was observed with Hitachi 7500 transmission electron microscope (TEM, Tokyo, Japan). The dynamic diameters of RPPs were determined by a Zetasizer Nano-ZS (Malvern Instruments, UK). The ultraviolet–visible–NIR absorbance spectra were recorded by ultraviolet–visible–NIR spectrophotometer (UV-3600, Shimadzu, Japan). And the loading efficiency and encapsulation efficiency of R848 was determined by absorbance at 324 nm. To evaluate the magnetic droplet vaporization performance of RPPs *in vitro*, approximately 1 mL SPIOs+RPPs nanoparticles suspension (24ug Fe and 1mg RPPs containing in 1ml PBS) placed in an eppendorf tube was irradiated with AMF, and an infrared thermal cameral (Fotric 226, Shanghai, China) was used for monitoring the temperature changes of SPIOs+RPPs to control the temperature at about 44 ℃. The phase transition of the RPPs was observed under the optical microscope (Leica, Germany). In addition, ultrasonography was performed before and after AMF irradiation respectively.

1. **Characterization of SPIOs：**

The morphology and size were observed through a field emission transmission electron microscope (FEI Tecnai G2, USA). The size distribution of SPIOs was counted and analyzed by Image J. The size distribution of SPIOs was counted and analyzed by Image J by randomly selecting several samples(n=150), measuring their sizes and statistically analyzing the results. The final result of the size of SPIOs was represented as mean±standard deviation(SD). Additionally, hydrodynamic diameters of SPIOs were also measured by DLS. The magnetic properties of SPIOs and SPIOs+RPPs were characterized by a Vibrating Sample Magnetometer (VSM) (BKT-4500). Under the irradiation of AMF, 0.5ml SPIOs and SPIOs+RPPs suspension at designed concentration placed in coil, the increasing temperature was recorded by the infrared thermal cameral, and temperature rising curves were plotted accordingly. The SAR value of SPIOs was calculated with the following formula: SAR = C(Vs/m) (dT/dt), where C was the volumetric heat capacity of solvent, Vs was the sample volume, m was the mass of nanoparticles, and dT/dt was the initial slope of the time-dependent temperature increase curve.

**2.3 *In vitro* toxicity assessment of SPIOs+RPPs:**

Standard CCK-8 viability assay was conducted to evaluate the *in vitro* cytotoxicity of SPIOs+RPPs. 4T1cells were pre-seeded in 96-well plates and co-incubated with SPIOs, RPPs, and SPIOs+RPPs at designed concentrations for 24h. Then, CCK-8 was added into the plates at a ratio of 1:10 to test the cell viabilities at a wavelength of 450 nm on a microplate reader a few minutes later.

**2.4 *In vitro* mild MHT of SPIOs+RPPs:**

4T1 cells were cultured in 60 mm dishes for 12h, and then cells were incubated with different samples, including saline, SPIOs, SPIOs+PPs, SPIOs+RPPs (Fe:24ug/ml, PPs or RPPs:200ug/ml) containing in 1ml PBS), and finally the medium was removed and cells were rinsed with PBS. For AMF-free groups, the cells were incubated with SPIOs, SPIOs+PPs, SPIOs+RPPs without AMF irradiation. For the AMF-stimulation groups, after incubated with SPIOs, SPIOs+PPs, SPIOs+RPPs, cells were irradiated with AMF (45 A, 500 kHz). After the dish was placed in the copper coil, the high-frequency induction heating equipment was turned on and the temperature of the solution was monitored by a thermal infrared imager during exposure to the AMF. The AMF was turned off once the temperature reached 44 ℃ and the AMF was turned on once the temperature reached 43 ℃, namely mild MHT. The mild MHT was conducted for 10 min.

**2.5 Cell Inhibition and Apoptosis Analysis:**

For a standard CCK-8 cell inhibition assay, 4T1 cells after above different treatments were seed in 96 wells plate. After different periods of culture, CCK-8 was added at a ratio of 1:10 into the wells to test the cell viabilities at a wavelength of 450 nm on a microplate reader after incubating for a period of time. For apoptosis analysis by flow cytometry, 4T1 cells after above different treatments were digested with trypsin and added 500 μL of PBS to redisperse. 5 μL of Annexin V-FITC and 5 μL of propidiumiodide (PI) were used to stain the live and dead cells for 15 min in the dark. Finally, cell apoptosis of cells after different treatments was examined using flow cytometry.

**2.6** **DAMPs Expression Analysis:**

Cells after above different treatments were cultured for another 8 h. For ELISA assay, the supernatant (serum-free) was collected for extracellular HGMB1 and ATP analysis using ELISA kits according to vendors’ protocols. For western blotting analysis, cells were rinsed with PBS for three times and RIPA cell lysis buffer was added. Equivalent amounts of proteins were loaded on a 10% sodium dodecyl sulfate-polyacrylamide gel (SDS-PAGE) and transferred to a nitrocellulose membrane. Firstly, the nitrocellulose membrane was incubated with the primary antibodies against HMGB1, ATP and ACTIN (Servicebio, China) at 4 °C overnight. Then, the protein bands were incubated with horseradish-peroxidase conjugated secondary antibody. The protein expression was visualized using a chemiluminescence system. For WB quantitative analysis, we measured integrated density value of the target strip by Alpha processing system, and calculated the ratio (the integrated density value of the target strip/ the integrated density value of ACTIN). And then, the ratio in control group was standardized as “one” to calculate the relative ratio of each group, represented the mean ±SD (n=3).

**2.7** **DC activation *in vitro*:**

Dendritic cells were isolated from the bone marrow of 6-8 week-old BALB/c mice according to an established method[4]. For *in vitro* DC activation experiments, DCs were treated with PBS, SPIOs, SPIOs+PPs and SPIOs+RPPs for 12 h. Alternatively, residues of 4T1 cells after mild MHT with either SPIOs, SPIOs+PPs or SPIOs+RPPs were also added into DC co-culture transwell system. After various treatments, DCs were stained with anti-CD11c FITC, anti-CD86 PE and anti-CD80 PC5.5 (BioLegend,USA) and then sorted by flow cytometry (Beckman Coulter Inc CytoFLEX S,USA).

**2.8 Anti-Tumor Model *In Vivo*:**

Female BALB/c mice (6 weeks old) were purchased from Enswell Biotechnology Ltd (Chongqing, China). All experimental protocols in this study were performed in the Chongqing Medical University Laboratory Animal Center and all protocols were approved by the Animal Ethics Committee of the Second Affiliated Hospital of Chongqing Medical University. For the primary tumor inoculation,4T1 cells (1×10^6^ cells per mouse) suspended in PBS were subcutaneously injected into the left of fifth mammary fat pad of each BALB/c mice.

**2.9 *In vivo* biosafety of SPIOs+RPPs:**

To evaluate the *in vivo* biosafety of SPIOs+RPPs, healthy BALB/c mice were intravenously administrated with 200ul SPIOs+RPPs nanospheres suspension (Fe:60ug, PPs or RPPs:400 ug). Mice were sacrificed pre the injection and at 3 d, 7 d, 14 d and 21 d (n=5) post the injection, and then blood samples were collected for hematology analysis and serum biochemical tests, respectively. Major organs (heart, liver, spleen, lung, and kidney) were subjected to H&E staining.

**2.10 *In vivo* mild MHT on 4T1 tumor bearing mice by SPIOs+RPPs:**

When the volume of tumor reached 70 mm^3^(volume calculated according to the formula V = length × width × width/2, the length and width were measured by digital calipers), mice were separated into seven groups with randomization. Mice injected with saline were set as control.

0.25mg SPIOs, SPIOs+PPs, and SPIOs+RPPs dispersed respectively in 50ul PBS (Fe:60ug, PPs or RPPs:400 ug) were prepared for intratumoral injection. For AMF-free groups, Balb/c mice were just intratumorally injected with SPIOs, SPIOs+PPs, SPIOs+RPPs without AMF irradiation. For the AMF stimulation groups, after intratumorally injected with SPIOs, SPIOs+PPs, SPIOs+RPPs, mice were irradiated with AMF (45 A, 500 kHz), and the temperature of tumors was maintained between 43 and 44 ℃ for 10 min. The temperature in tumor site was monitored by a thermal infrared camera during exposure to the AMF. The mild MHT was just performed once during the whole process of treatment.

**2.11 Immunofluorescence of CRT, HMGB1 and Tumor Pathological Slices *in Vivo:***

The mice were sacrificed at day 3 and the tumors were dissected. Then tumors were mounted with OCT compound. 5 µm slices were separated by microtone, the slices were blocking–washing–incubating–staining according to standard procedures. Last imaging was carried out by Fluorescent microscope. The percent of the released HMGB1 and CRT exposure were calculated by measuring the fluorescent intensity.

**2.12** **Assessment of DC maturation *in vivo*:**

To study the activation of DCs *in vivo*, three days after diverse treatments, the inguinal lymph nodes around the primary tumor of 4T1 tumor-bearing Balb/c mice in above seven groups were collected. The maturity of DCs in the lymph nodes was then examined by flow cytometry after immunofluorescence staining anti-CD11c FITC, anti-CD86 PE and anti-CD80 PC5.5 (BioLegend, USA) antibody according to the procedure of the manufacturer and then sorted by flow cytometry.

**2.13 Cytokine Assay:**

Serum samples were isolated from mice after various treatments and diluted for analysis. Tumour necrosis factor (TNF-a, Dakewe biotech), interferon gamma (IFN-γ, Dakewe biotech), IL-12 (Dakewe biotech) and IL-6 (Dakewe biotech) were detected with ELISA kits according to manufacturers’ instructions.

**2.14 *In Vivo* Distant Metastatic Tumor Inhibition:**

To mimic distant metastasis, 4T1 cells (2×10^5^) suspended in PBS were subcutaneously injected into the right of fifth mammary fat pad 7 days later after primary tumor inoculation in the left mammary fat pad. Mice were separated into seven groups with randomization: (1) Saline, (2) SPIOs, (3) SPIOs+PPs, (4) SPIOs+RPPs, (5) SPIOs+AMF, (6) SPIOs+PPs+AMF, (7) SPIOs+RPPs+AMF. For the primary tumor on the left mammary fat pad of mice, SPIOs, SPIOs+RPPs were intratumorally injected in the 2^nd^ and 5^th^ group, SPIOs+PPs were intratumorally injected in the 3^rd^ and 6^th^ group, SPIOs+RPPs were intratumorally injected in the 4^th^ and 7^th^ group. And then AMF was applied in the 5^th^, 6^th^ and 7^th^ group to realize mild MHT subsequently. The detailed methods were same as the mentioned above. The growth of primary tumor and distant metastatic tumor as well as the body weights of each mouse were recorded by vernier calipers every three days. To evaluate the T cell response in secondary tumors, tumors were harvested from mice in different groups and stained with anti-CD3-FITC, anti-CD8a-APC, anti-CD4-PE (Biolegend, USA) antibodies according to the manufacturer’s protocols.

**2.15** ***In Vivo* Lung metastasis Inhibition:**

To establish lung metastases, 4T1 cells (2×10^5^) were administered intravenously via tail vein infusion into each BALB/c mouse 7 days later after primary tumor inoculation. Mice were separated into seven groups with randomization, and treated by different methods. The details were same as the mentioned above. Mice were sacrificed at day 21, and the lung tissues were taken out to count the number of tumor metastasis foci after stained with Bouin’s solution and for H&E staining histopathological analysis.

**2.16** ***In vitro* and *in vivo* US imaging**

An ultrasound imaging system (Vevo LAZR, Canada) was used to evaluate the MDV capability and the ultrasound imaging performance of SPIOs+RPPs. The US imaging mode parameters were fixed (frequency: 18 MHz; power: 4%; contrast gain: 43 dB; 2D gain: 22 dB; dynamic range: 35 dB). The *in vitro* groups were as follows: PBS, SPIOs, SPIOs-RPs (10mg ml^-1^), SPIOs+RPPs (5mg ml^-1^,10 mg ml^-1^,15 mg ml^-1^) aqueous solution(n=3). Each group irradiated with a fixed power-parameter AMF (500 kHz; 45 A) and the US imaging was immediately completed at both B-mode and CEUS mode. Finally, DFY software (Chongqing Medical University, Chongqing, China) was used to quantitate the echo intensity of the ROI. For *in vivo* US imaging evaluation, SPIOs, SPIOs+RPs, and SPIOs+RPPs (10 mg mL^-1^,50ul) were intratumorally injected into each 4T1 tumor bearing mouse (n=3), and mice injected with saline as control. The US images of tumor at both B-mode and CEUS mode were recorded immediately after AMF irradiation (500 kHz; 45 A; 10 min). The analysis of the echo intensity of the ROI was acquired through DFY software.

**Supporting Figure and caption**

**
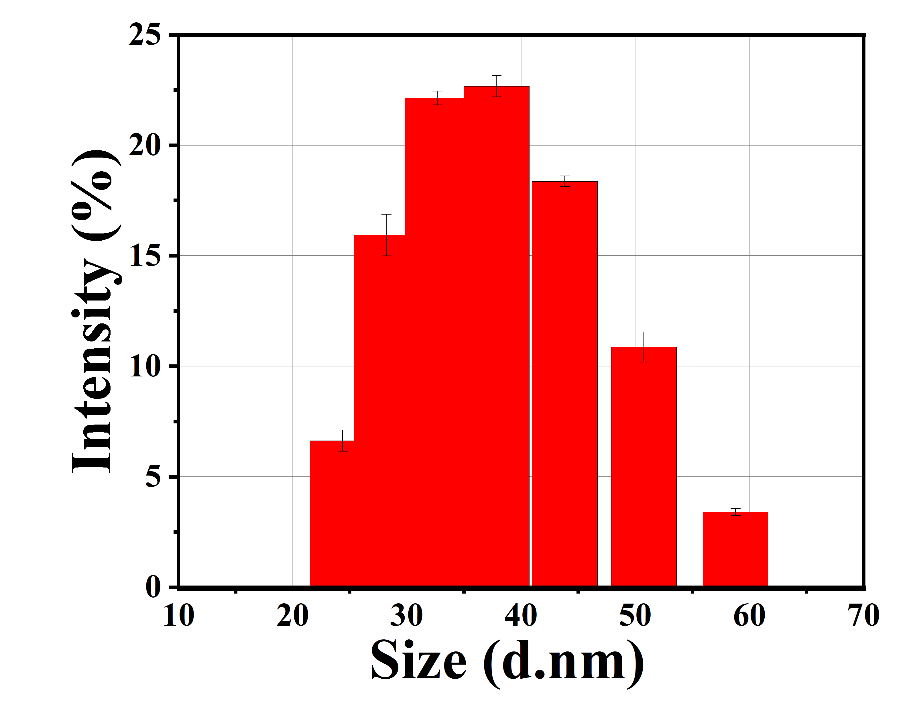
**

**Figure S1 Hydrodynamic size distribution of RPPs by DLS.**

**
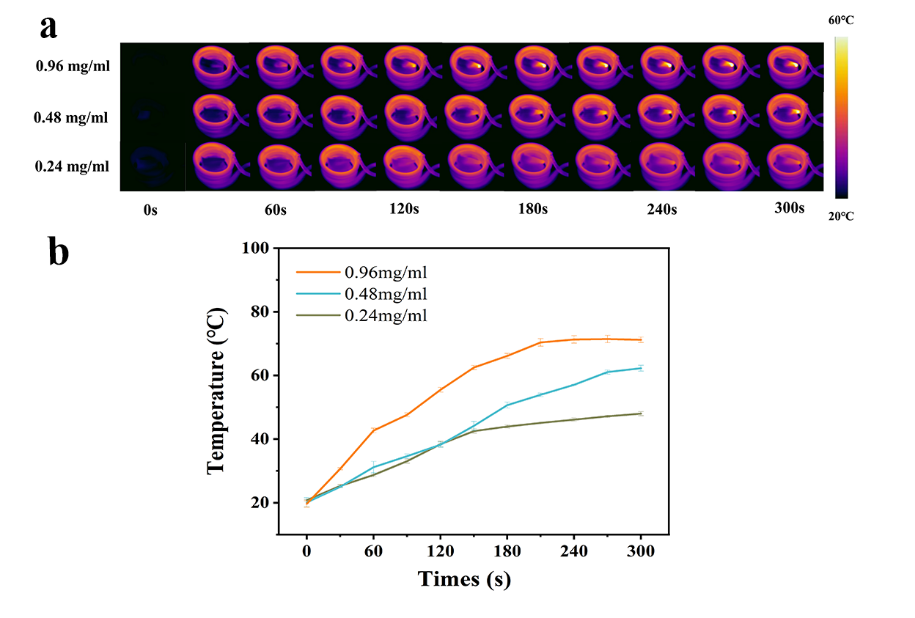
**

**Figure S2.** **The magnetothermal effect of the nanosystem.**

a) Real -time *in vitro* IR thermal imaging of SPIOs at different concentrations.

b) Quantitative temperature risingrise curves of SPIOs at different concentrations.


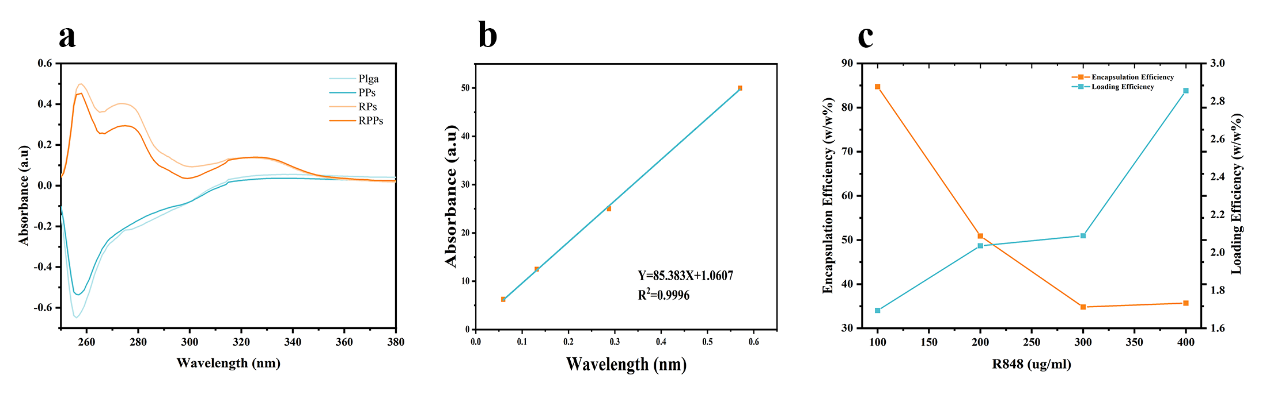


**Figure S3 UV−vis absorbtion spectra of RPPs and the encapsulation efficiency and loading efficiency of RPPs.**

a)UV−vis absorption spectra of free blank PLGA、RPPs、RPs and PPs.

b) standard curves of R848. c) The encapsulation efficiency and loading efficiency of R848 at different feeding concentration. The concentration of R848 is fixed at 200 µg.


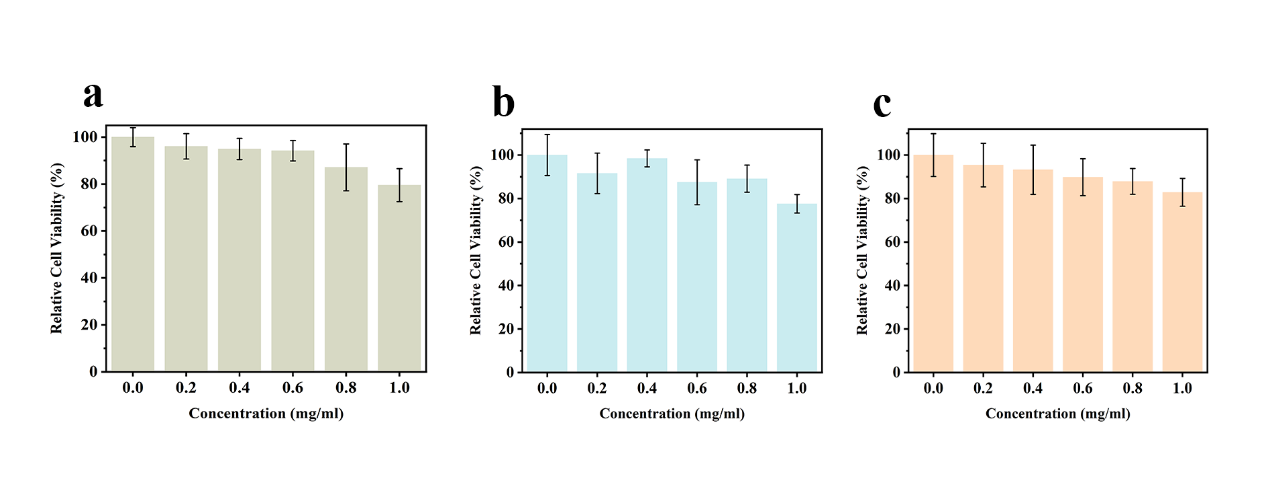


**Figure S4 Cells viabilities after incubation with SPIOs, RPPs and SPIOs+RPPs.**

a, b, c) 4T1 cells viabilities after incubation with SPIOs, RPPs and SPIOs+RPPs respectively estimated through standard CCK-8 assay.


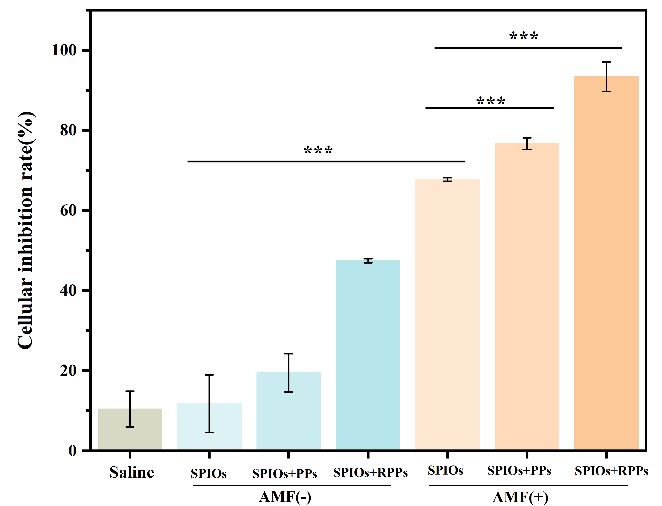


**Figure S****5 CCK-8 assay of Cellular inhibition rate after different treatments.**

Cellular inhibition rate of 4T1 cells after different treatments estimated by CCK-8 assay. (one-way ANOVA with Tukey’s post-hoc test, ***P < 0.001）


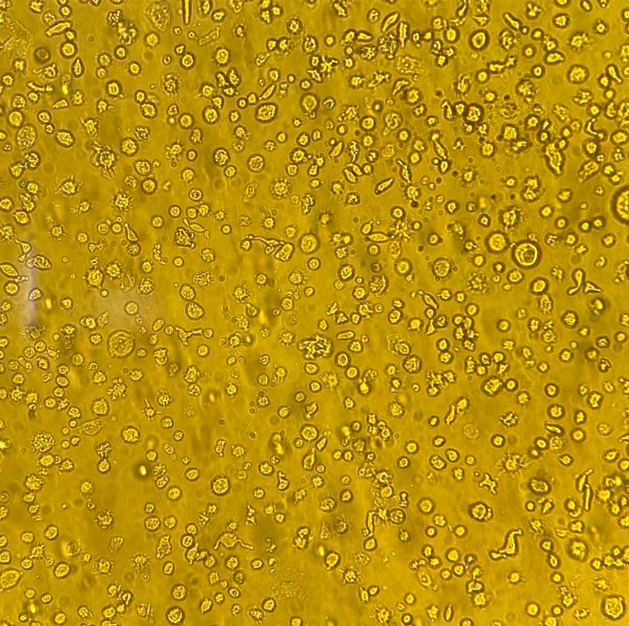


**Figure S6 Morphology of BMDCs under optical microscope.**

Morphology of BMDCs after 10 days’ cultivation by the GM-CSF and IL-4 under optical microscope. Numerous non-adherent mature DCs with projections are visible. (Original magnification 200×).

**
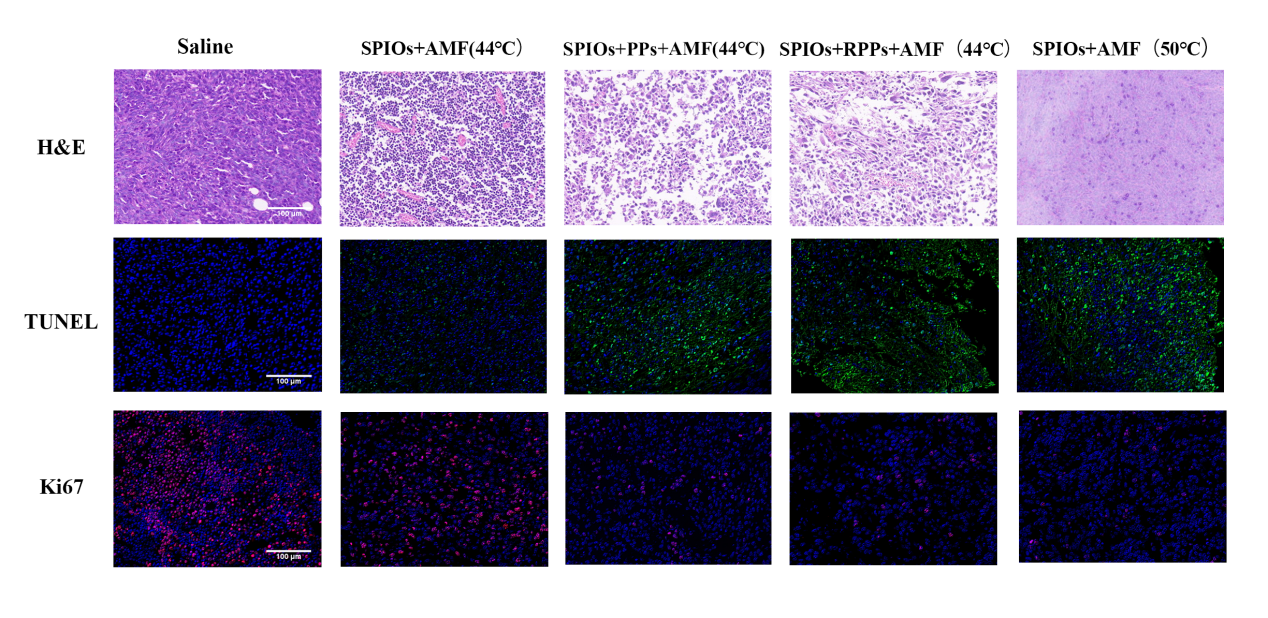
**

**Figure S7** **Representative images of H&E staining, TUNEL, and Ki67 immunostaining of tumor slices after different trentments (scale bars=50μm).**


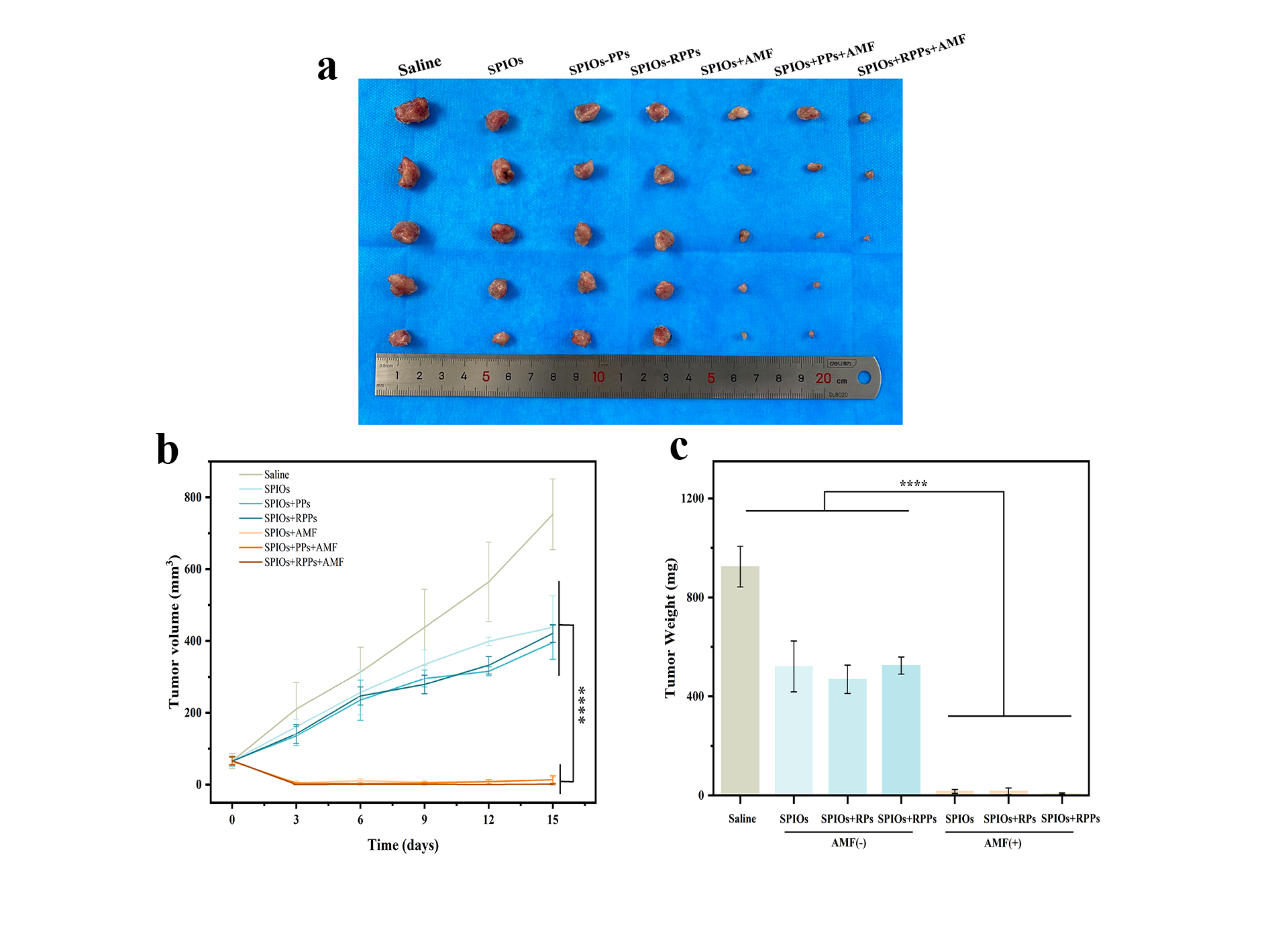


**Figure S8 The analysis of tumor growth after different treatments.**

a) Photographs of excised tumors at the end point of different treatment groups. b) Tumor volume curves of the tumors in diferent groups (n=5). c)The weight analysis of excised tumors at the end point of different treatment groups(n=5). （one-way ANOVA with Tukey’s post-hoc test, ****p < 0.0001）


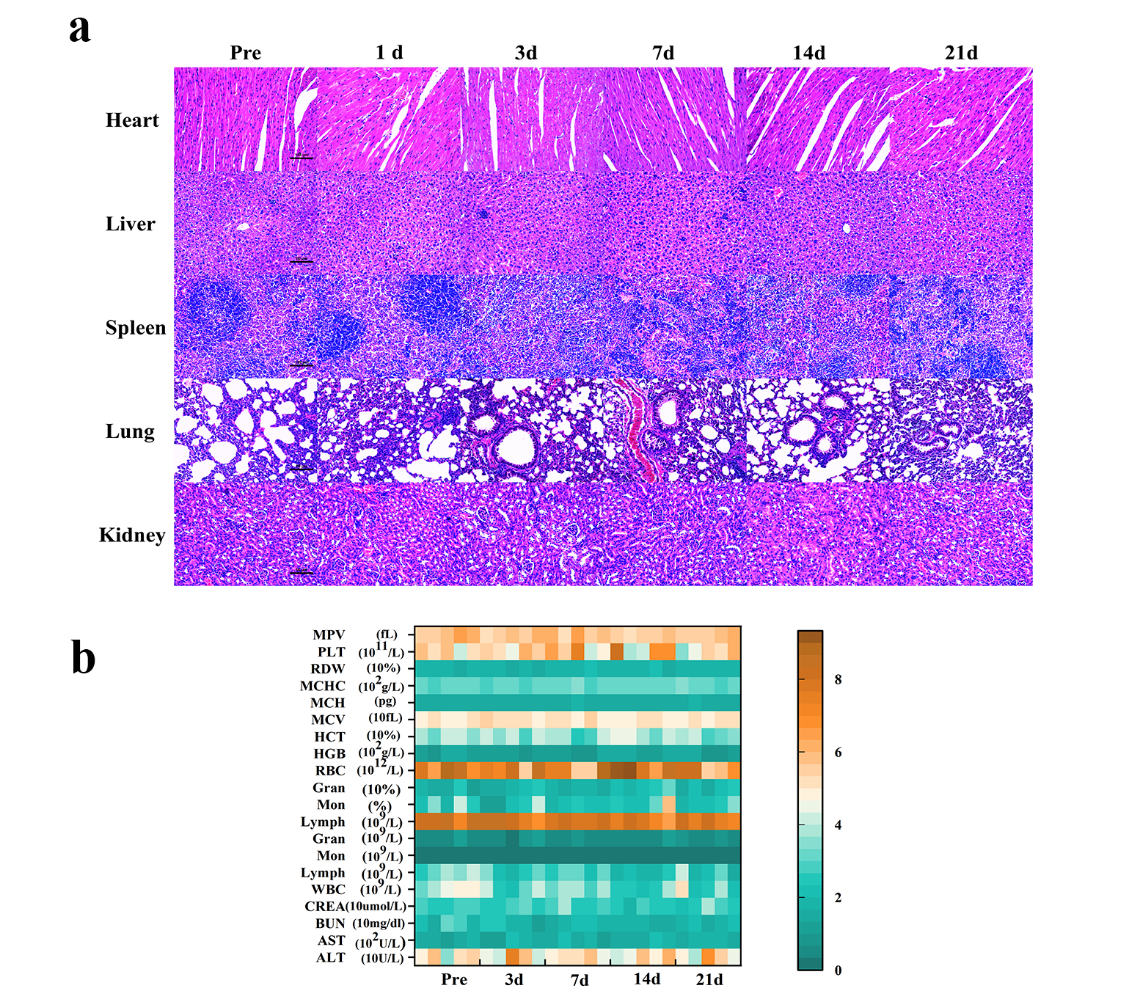


**Figure S9 Biosafety analysis of SPIOs+RPPs *in vivo*.**

a) Histological analysis of major organs (heart, liver, spleen, lung, and kidney) in BALB/c mice with differnernt treatments (Scale bar: 200 μm) b) The blood biochemical and routine blood test of mice after intravenous administration of SPIOs+RPPs at different time points.


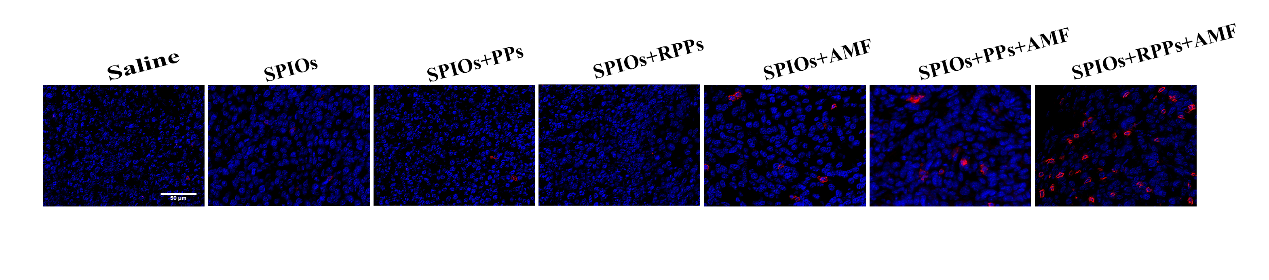


**Figure S10 CD8+ immunostaining of distant metastatic tumor slices after different treatments.**

1. Abraham E, Minoshima K, Matsumoto H: Femtosecond laser-induced breakdown in water: time-resolved shadow imaging and two-color interferometric imaging. *Optics Communications* 2000, 176(4-6):441-452.

2. Guo Y, Ran Y, Wang Z, Cheng J, Cao Y, Yang C, Liu F, Ran H: Magnetic-responsive and targeted cancer nanotheranostics by PA/MR bimodal imaging-guided photothermally triggered immunotherapy. *Biomaterials* 2019, 219:119370.

3. Liu K, Han L, Tang P, Yang K, Gan D, Wang X, Wang K, Ren F, Fang L, Xu Y: An anisotropic hydrogel based on mussel-inspired conductive ferrofluid composed of electromagnetic nanohybrids. *Nano Letters* 2019, 19(12):8343-8356.

4. Pan J, Hu P, Guo Y, Hao J, Ni D, Xu Y, Bao Q, Yao H, Wei C, Wu Q: Combined magnetic hyperthermia and immune therapy for primary and metastatic tumor treatments. *ACS nano* 2020, 14(1):1033-1044.
